# Factors associated with healthy aging in Latin American populations

**DOI:** 10.1038/s41591-023-02495-1

**Published:** 2023-08-10

**Authors:** Hernando Santamaria-Garcia, Agustín Sainz-Ballesteros, Hernán Hernandez, Sebastian Moguilner, Marcelo Maito, Carolina Ochoa-Rosales, Michael Corley, Victor Valcour, J. Jaime Miranda, Brian Lawlor, Agustin Ibanez

**Affiliations:** 1 Global Brain Health Institute, University of California San Francisco, San Francisco, CA USA; 2 Center of Memory and Cognition Intellectus, Hospital Universitario San Ignacio Bogotá, San Ignacio, Colombia; 3 Pontificia Universidad Javeriana (PhD Program in Neuroscience) Bogotá, San Ignacio, Colombia; 4 Latin American Brain Health Institute, Universidad Adolfo Ibañez, Santiago de Chile, Chile; 5 Faculty of Engineering, University of Concepción, Concepción, Chile; 6 Cognitive Neuroscience Center, Universidad de San Andrés and Consejo Nacional de Investigaciones Científicas y Técnicas, Buenos Aires, Argentina; 7 Department of Neurology, Massachusetts General Hospital and Harvard Medical School, Boston, MA USA; 8 Department of Medicine, Division of Infectious Diseases, Weill Cornell Medicine, New York, NY USA; 9 Memory and Aging Center, University California San Francisco, San Francisco, CA USA; 10 Centro de Excelencia en Enfermedades Crónicas, Universidad Peruana Cayetano Heredia, Lima, Peru; 11 Department of Medicine, School of Medicine, Universidad Peruana Cayetano Heredia, Lima, Peru; 12 Faculty of Epidemiology and Population Health, London School of Hygiene and Tropical Medicine, London, UK; 13 The George Institute for Global Health, University of New South Wales, Sydney, New South Wales Australia; 14 Trinity College Dublin, The University of Dublin, Dublin, Ireland

**Keywords:** Neurodegenerative diseases, Psychology

## Abstract

Latin American populations may present patterns of sociodemographic, ethnic and cultural diversity that can defy current universal models of healthy aging. The potential combination of risk factors that influence aging across populations in Latin American and Caribbean (LAC) countries is unknown. Compared to other regions where classical factors such as age and sex drive healthy aging, higher disparity-related factors and between-country variability could influence healthy aging in LAC countries. We investigated the combined impact of social determinants of health (SDH), lifestyle factors, cardiometabolic factors, mental health symptoms and demographics (age, sex) on healthy aging (cognition and functional ability) across LAC countries with different levels of socioeconomic development using cross-sectional and longitudinal machine learning models (*n* = 44,394 participants). Risk factors associated with social and health disparities, including SDH (*β* > 0.3), mental health (*β* > 0.6) and cardiometabolic risks (*β* > 0.22), significantly influenced healthy aging more than age and sex (with null or smaller effects: *β* < 0.2). These heterogeneous patterns were more pronounced in low-income to middle-income LAC countries compared to high-income LAC countries (cross-sectional comparisons), and in an upper-income to middle-income LAC country, Costa Rica, compared to China, a non-upper-income to middle-income LAC country (longitudinal comparisons). These inequity-associated and region-specific patterns inform national risk assessments of healthy aging in LAC countries and regionally tailored public health interventions.

## Abstract (web summary)

Machine learning models showed that social disparities, cardiometabolic disease and mental health were the main predictors of aging in Latin American populations, with these factors being more pronounced in low- and middle-income compared to high-income Latin American countries.

## Main

Aging is not a uniform process across the world. Most of the research into cognitive and functional aging has traditionally been conducted in high-income settings within the United States and Europe, neglecting diverse populations and the specific combination of risk factors seen in Latin American and Caribbean (LAC) countries. Despite the urgent need to assess regional diversity and deliver tailored evidence for diverse populations^1–4^, the evidence around healthy aging in LAC countries is lacking. Addressing this knowledge gap is essential because risks stem from multiple disparity-related cumulative exposures affecting aging and dementia. Latin American populations have unique ethnic admixtures, education and sociodemographic heterogeneity. In addition, the current prevalence of dementia in LAC countries is estimated at 8.5% and is projected to be 19.33% by 2050, representing an increase of 220% approximately^5^. Such prevalence is higher compared to other regions^5^, including Europe (currently 6.9% and projected up to 7.7% by 2050) or North America (currently 6.5% and projected up to 12.1% by 2050)^5,6^. Previous studies in high-income countries (HICs)^1,7^ that capture risk factors of healthy and pathological brain aging were not accurate for low-income and middle-income countries (LMICs) or LAC countries^1,6^. Thus, assessing specific risk factors in LAC countries constitutes a critical priority for understanding healthy aging.

Healthy brain aging refers to the functional brain ability that allows someone to live their life to their fullest capacity. Healthy aging is traditionally evaluated using cognitive and functional ability measures, constituting proxy markers of brain health^8^. Cognition involves multiple domains (that is, attention, problem-solving, learning and memory, among others), while functional ability encompasses personal activities of daily living (ADLs) and higher-order instrumental skills^8^.

Several factors have been associated with healthy or unhealthy aging outcomes in previous studies^3,9^. These include demographic factors such as age and sex; social factors such as educational level, socioeconomic status (SES) and social support, together known as social determinants of health (SDH); health status (including cardiometabolic factors such as hypertension, diabetes and obesity, and falls); mental health symptoms (including depression and anxiety); and lifestyle factors (alcohol consumption, smoking and physical activity). Most of those factors have been described as potentially modifiable risk factors for dementia by the Lancet Commission on Dementia Prevention, Intervention, and Care^3^. Although multiple factors contribute to healthy and pathological aging in HICs, less-modifiable factors, such as age^10–12^ and sex^13,14^, are considered top contributors to pathological aging^10–12^. Healthy aging in HICs is also influenced by modifiable factors such as cardiometabolic factors, mental health symptoms and lifestyle factors^5,15^. The relative importance of mentioned risk factors may be different and heterogenous in countries with increased social and health disparities such as LAC countries^3,16^. Compared to HICs, in LAC countries converging multiple factors were associated with pathological aging, including a substantial contribution of SDH^17^, a higher prevalence of cardiometabolic factors^18^, mental health symptoms^19^ and barriers to healthy lifestyle^20,21^.

Previous evidence in healthy aging in LAC countries identified multiple gaps^1,22–25^, including insufficient understanding of the unique determinants and risk factors of aging in the region, and no attempt to simultaneously assess the associations and interactions between the different potential risk factors of healthy aging. Omissions in evaluating such interactions can lead to spurious or improper relationships between risk factors and healthy aging outcomes. Moreover, emerging evidence suggests that models of risk do not always generalize from HICs to LMICs^1^. Other research gaps in the region include limited studies combining cross-sectional and longitudinal methods; absence of automated data-driven approaches for assessing multiple risk and protective factors, with no inclusion of techniques to confirm the validity of results (that is, out-of-sample validation procedures), leading to potential biases due to the assumptions of a priori theoretical models; inadequate representation of diverse populations from HICs, upper-income and middle-income countries (UMICs), and LMICs; and lack of region-specific risk factors of cognition and functional ability. Our work aimed to address these gaps using a convergent approach.

Using cross-sectional and longitudinal approaches, we assessed multiple potential risk factors (demographic, SDH, health status, lifestyle and mental health symptoms) of cognition and functional ability in healthy aging across LAC countries. We hypothesized that disparity-related social and health factors, as opposed to demographic factors such as age and sex, would be more important in healthy aging across LACs compared to data reported in previous studies from HICs. We also expected country-level differences in healthy aging risk factors in LAC countries according to their income categories.

## Results

**Fig. 1:**
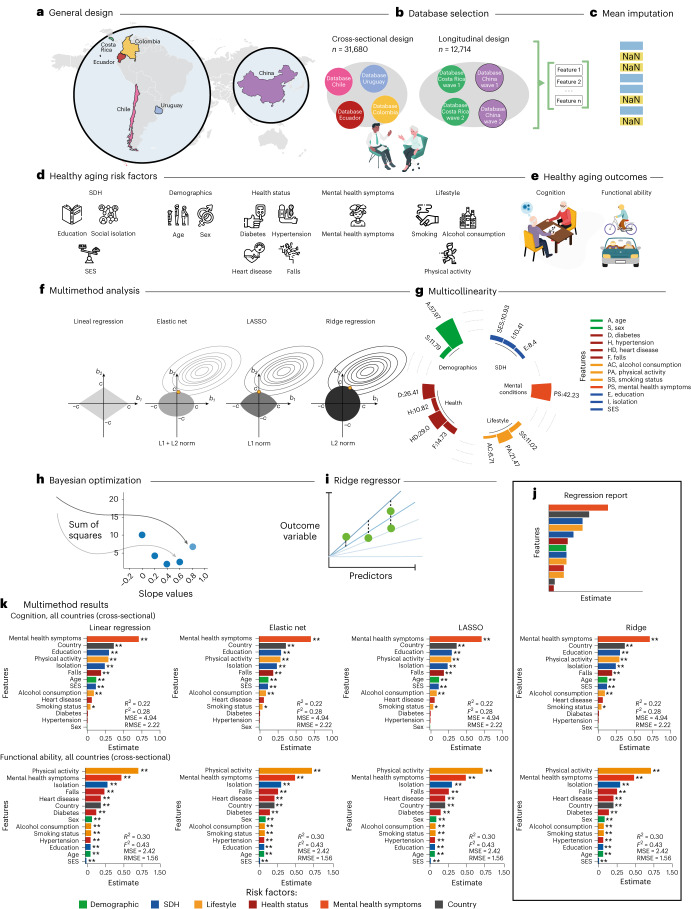
Methodological framework. **a**, General design of the study describing the countries included in the cross-sectional analyses (Chile, Uruguay, Colombia and Ecuador) and in the longitudinal analyses (Costa Rica and China). **b**, Database selection in the cross-sectional (*n* = 31,680 participants) and longitudinal (*n* = 9,258 participants) studies (total *n* = 40,938 participants). **c**, Imputation procedures. **d**, Risk factors, including demographics, SDH, health status (cardiometabolic factors and falls), mental health symptoms and lifestyle risk factors. **e**, Outcomes: cognition and functional ability. **f**, Multimethod approach, including different regressions (linear regression, elastic net, LASSO, ridge regression). **g**, Multicollinearity between risk factors that justified the selection of ridge regression as the adequate model. **h**, Bayesian optimization to find the best hyperparameters for ridge regression. **i**, Regression step used with the ridge regression. **j**, Regression report. **k**, The multimethod results revealed high consistency across methods using goodness-of-fit metrics (*R*^2^, Cohen’s *F*_2_, MSE and RMSE) and a high coherence in the weight and ranking of risk factors of healthy aging (*β* estimates). In **k**, the upper panel presents the multimethod findings related to cognitive performance. The lower panel displays the functional ability data across all countries in Latin America. Risk factors: demographics, SDH, health, lifestyle, mental health symptoms and country.

**Extended Data Fig. 1:**
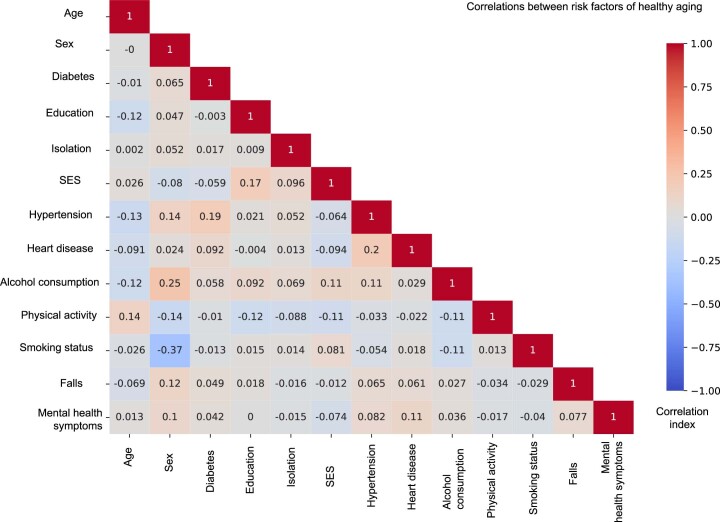
Correlations between factors and outcomes associated with healthy aging. Matrix of correlation between factors and outcomes (cognition and functional ability) of healthy aging.

Using a data-driven approach based on machine learning procedures, we assessed multiple potential risk factors (including demographic factors, SDH, health status, mental health symptoms and lifestyle) that affect cognition and functional ability in healthy aging (total *n* = 44,394 participants; Fig. 1a–e). Cross-sectional and longitudinal data from nationally representative survey cohorts included various LACs with different levels of socioeconomic development (Fig. 1b). As a first step, a multimethod approach (Fig. 1f) comprising linear regression, elastic net, least absolute shrinkage and selection operator (LASSO), and ridge regression was implemented to ensure the robustness of our machine learning results. We choose ridge regression based on the convergence observed with this multimethod approach (Extended Data Tables 1 and 2, and Extended Data Fig. 1). We used harmonized databases from national aging surveys from four LACs with different socioeconomic indexes based on the World Data Bank categorization. Two countries ranked as Latin American-LMICs (LA-LMICs, Colombia (*n* = 23,694 participants) and Ecuador (*n* = 5,235 participants)) and two countries as LA-HICs (Chile (*n* = 1,301 participants)) and Uruguay (*n* = 1,450 participants) were incorporated. We ran a second longitudinal analysis (*n* = 9,258 participants) from Costa Rica (*n* = 5,694 participants), a UMIC (LA-UMIC), and compared it with a non-LA-UMIC (China, *n* = 3,564 participants), which has the same socioeconomic category.

### Cross-sectional analyses in LAC countries

We first assessed the main risk factors of cognition and functional ability using cross-sectional national surveys from all LAC countries. We then used the same approach focusing on LA-LMICs versus LA-HICs and finally across different countries. The multimethod results revealed consistency across methods and metrics (*R*^2^, Cohen’s *F*_2_, mean square error (MSE), root mean square error (RMSE)) and a high coherence in the weight and ranking of risk factors (by using *β* estimates) of healthy aging in all countries (Fig. 1k).

**Fig. 2:**
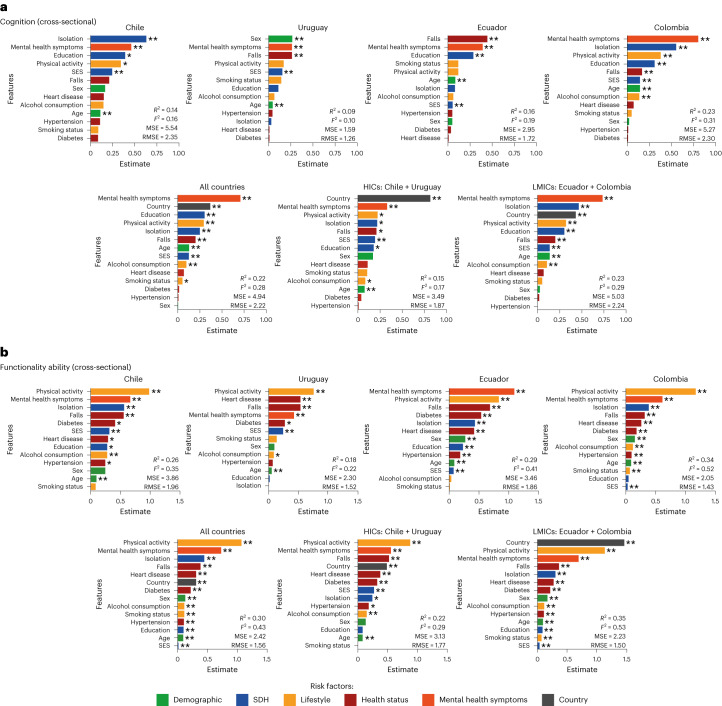
Cross-sectional results of cognition and functional ability in Latin America. **a**,**b**, Results for cognition (**a**) and functional ability (**b**) for each country, all collapsed countries, as well as LA-HICs (Chile and Uruguay) and LA-LMICs (Ecuador and Colombia). The *R*^2^, Cohen’s *F*_2_, MSE and RMSE are reported for each model. The feature importance and their statistical significance are also provided for each model. *β* estimates were used to assess the weight of each feature in the models. The risk factors are: demographics, SDH, lifestyle, health status (cardiometabolic factors and falls), mental health symptoms and country. The two-sided *P* value of a Student’s *t*-statistic was calculated for the *β* values of the regression. *P < 0.05; **P < 0.01. No asterisk means not significant.

A model assessing the risk factors of cognition from all LAC countries was significant (*F*_1_, _28,109_ = 565.61, *P* < 0.0001, *R*^2^ = 0.22 ± 0.026, *F*_2_ = 0.28, MSE = 4.94, RMSE = 2.42) (Fig. 2a). The most important risk factors in order of relevance were: mental health symptoms (*β* = 0.71, *P* < 0.0001), country (*β* = −0.37, *P* < 0.01), physical activity (*β* = −0.3, *P* < 0.0001), education (*β* = 0.3, *P* < 0.0001), isolation (*β* = 0.25, *P* < 0.0001), age (*β* = −0.13, *P* < 0.0001) and SES (*β* = 0.13, *P* < 0.0001). Moreover, alcohol consumption (*β* = −0.11, *P* < 0.01) and smoking (lifestyle, *β* = −0.05, *P* < 0.05) were also significant. No significant effects of cardiometabolic factors were observed for cognition (Table 1 and Fig. 2a).

**Table 1:** Demographic and healthy aging information across studies

Country	Sex		Age		Education		Cognition (MMSE)^a^		Functional ability (Barthel)	
	Category	*n*	Mean	s.d.	Mean	s.d.	Mean	s.d.	Mean	s.d.
**Demographic and general information of the cross-sectional analyses**										
Chile	Female	855	72.13	8.3	1.09	0.38	11.69	3.1	6.93	2.42
	Male	446	70.51	7.4	1.18	0.56	12.11	2.69	7.83	2.11
Uruguay	Female	920	71.07	7.64	1.15	0.47	12.95	1.53	7.75	1.81
	Male	530	70.72	6.81	1.27	0.64	12.98	1.17	8.04	1.54
Colombia	Female	13,582	70.8	8.26	1.08	0.36	11.82	2.74	8.01	1.84
	Male	10,112	70.84	8.12	1.12	0.44	11.94	2.53	8.4	1.65
Ecuador	Female	2,767	71.48	8.72	1.08	0.38	11.44	2.35	6.58	2.3
	Male	2,468	71.3	8.45	1.14	0.51	11.76	2.21	7.31	2.2
**Demographic and general information of the longitudinal analyses**										
Costa Rica	Female	1109	59.5	3.1	1.5	0.7	10.9	1.2	8.03	1.3
	Male	642	59.5	3.2	1.5	0.7	10.6	1.3	8.33	1.2
China	Female	2194	83.1	10.6	1.0	0.2	10.0	4.4	7.6	2.9
	Male	2093	79.8	9.2	1.2	0.4	11.4	3.7	8.5	2.5

MMSE, Mini-Mental State Examination.

^a^For the longititudinal analyses, the cognition and funtional ability relate to wave 2.

The model predicting functional ability was significant (*F*_1, 27,991_ = 866.71, *P* < 0,00001; *R*^2^ = 0.3 ± 0.026, *F*_2_ = 0.43, MSE = 2.42, RMSE = 1.56). In order of importance, the most relevant risk factors were: physical activity (*β* = −1.06, *P* < 0.0001), mental health symptoms (*β* = 0.73, *P* < 0.0001), isolation (*β* = −0.44, *P* < 0.0001), falls (*β* = 0.38, *P* < 0.0001), country (*β* = 0.33, *P* < 0.0001), heart disease (*β* = 0.29, *P* < 0.0001), diabetes (*β* = 0.22, *P* < 0,0001), sex (*β* = 0.13, *P* < 0.0001), education (*β* = 0.11, *P* < 0.001), alcohol consumption (*β* = 0.11, *P* < 0.0001), smoking (*β* = −0.1, *P* < 0.001), hypertension (*β* = 0.09, *P* < 0.001), age (*β* = −0.09, *P* < 0.0001) and SES (*β* = 0.01, *P* < 0.01; Table 2 and Fig. 2b).

**Table 2:** Comparisons of the risk factors of aging between LA-HICs and LA-LMICs in cross-sectional analyses using the Ridge regression models

Risk factor	Feature	LA-HICs			LA-LMICs			LA-HICs versus LA-LMICs
		*β* estimates	*t*	*P*	*β* estimates	*t*	*P*	
**Comparison of risk factors of cognition between LA-HICs and LA-LMICs**								
	Intercept	14.35	16.72	<0.0001	17.59	64.49	<0.0001	–
	Country	0.81	10.23	<0.0001	0.43	8.59	<0.0001	LA-HICs > LA-LMICs *P* < 0.0001
Demographics	Age	−0.08	−61.16	<0.0001	−0.13	−3.74	<0.0001	LA-HICs < LA-LMICs *P* < 0.0001
	Sex	−0.18	−1.99	NS	0.02	0.7	NS	–
SDH	Isolation	0.22	2.12	<0.05	0.46	11.13	<0.0001	LA-HICs < LA-LMICs *P* < 0.0001
	SES	0.19	4.54	<0.0001	0.13	22.53	<0.0001	LA-HICs < LA-LMICs *P* < 0.0001
	Education	0.18	2.32	<0.05	0.3	8.67	<0.0001	LA-HICs < LA-LMICs *P* < 0.0001
Health status	Diabetes	0.04	0.35	NS	0.01	0.4	NS	–
	Hypertension	0.0	0.06	NS	0	0.01	NS	–
	Heart disease	−0.11	−1.22	NS	−0.07	−1.65	NS	–
	Falls	0.21	2.53	<0.05	0.2	6.61	<0.0001	LA-HICs < LA-LMICs *P* < 0.001
Mental health	Mental health symptoms	0.34	3.45	<0.001	0.73	15.46	<0.0001	LA-HICs < LA-LMICs *P* < 0.0001
Lifestyle	Physical activity	−0.23	−2.41	<0.05	−0.32	−9.12	<0.0001	LA-HICs < LA-LMICs *P* < 0.0001
	Alcohol consumption	0.08	2.1	<0.05	0.1	3.09	<0.01	–
	Smoking status	−0.11	−1.34	NS	−0.05	−1.69	NS	–

**Comparison of risk factors of functional ability across LA-HICs and LA-LMICs**								
	Intercept	7.25	8.59	<0.0001	9.81	54.06	<0.0001	–
	Country	0.49	6.28	<0.0001	1.46	44.13	<0.0001	LA-HICs < LA-LMICs *P* < 0.0001
Demographics	Sex	0.13	1.46	NS	0.16	7.92	<0.0001	NS
	Age	−0.08	−63.59	<0.0001	−0.09	−389.92	<0.0001	NS
SDH	SES	0.27	6.65	<0.0001	0.04	9.0	<0.0001	LA-HICs < LA-LMICs *P* < 0.0001
	Isolation	0.24	2.29	<0.05	0.3	10.86	<0.0001	LA-HICs < LA-LMICs *P* < 0.05
	Education	0.08	1.04	NS	0.08	3.57	<0.001	NS
Health status	Diabetes	0.33	2.95	<0.01	0.22	8.71	<0.0001	LA-HICs < LA-LMICs *P* < 0.0001
	Hypertension	0.18	2.35	<0.01	0.11	5.6	<0.0001	LA-HICs < LA-LMICs *P* < 0.0001
	Heart disease	0.37	4.38	<0.0001	0.27	10.25	<0.0001	LA-HICs < LA-LMICs *P* < 0.0001
	Falls	0.52	6.34	<0.0001	0.36	18.31	<0.0001	LA-HICs < LA-LMICs *P* < 0.01
Mental health	Mental health symptoms	0.55	5.75	<0.0001	0.69	22.01	<0.0001	LA-HICs < LA-LMICs *P* < 0.05
Lifestyle	Physical activity	−0.87	−8.93	<0.0001	−1.13	−48.55	<0.0001	LA-HICs < LA-LMICs *P* < 0.0001
	Alcohol consumption	0.15	3.7	<0.001	0.11	5.19	<0.0001	NS
	Smoking status	0.0	0.02	NS	−0.07	−3.49	<0.001	NS

NS, not significant. The two-sided *P* value of a Student’s *t*-test was calculated for the *β* estimates of the regression.

### Stratification according to country income level

For LA-LMICs, the model predicting cognition was significant (*F*_1, 25,769_ = 535.21, *P* < 0.00001; *R*^2^ = 0.23 ± 0.0145, *F*_2_ = 0.29, MSE = 5.03, RMSE = 2.24). The most relevant risk factors in the model were: mental health symptoms (*β* = 0.73, *P* < 0.0001), isolation (*β* = 0.46, *P* < 0.0001), country (*β* = 0.43, *P* < 0.0001), physical activity (*β* = −0.32, *P* < 0.0001), education (*β* = 0.30, *P* < 0.0001), falls (*β* = 0.2, *P* < 0.0001), SES (*β* = 0.13, *P* < 0.0001), age (*β* = 0.-13, *P* < 0.0001) and alcohol consumption (*β* = 0.08, *P* < 0.01).

For LA-HICs, the model predicting cognition was also significant (*F*_1, 2,340_ = 28.82, *P* < 0.00001; *R*^2^ = 0.15 ± 0.0672, *F*_2_ = 0.17, MSE = 3.49, RMSE = 1.87) (Fig. 2a). The most relevant risk factors included: country (*β* = 0.81, *P* < 0.0001), mental health symptoms (*β* = 0.34, *P* < 0.001), physical activity (*β* = −0.23, *P* < 0.05), isolation (*β* = 0.22, *P* < 0.05), falls (*β* = 0.21, *P* < 0.05), SES (*β* = 0.19, *P* < 0.0001), education (*β* = 0.18, *P* < 0.05), sex (*β* = 0.18, *P* < 0.05), alcohol consumption (*β* = 0.08, *P* < 0.05) and age (*β* = −0.08, *P* < 0.0001).

We compared the weight of each risk factor of cognition in the models of LA-LMICs and LA-HICs based on the effect sizes (*F*_2_). We observed that SES, isolation, mental health symptoms, physical activity and falls have coefficient estimates higher in LMICs than in HICs. Moreover, age and sex had a more significant role in LA-HICs than in LA-LMICs. Cardiometabolic factors did not reach significant values neither in LA-LMICs nor in LA-HICs (Table 2 and Fig. 2a).

The model predicting functional ability was significant for LA-LMICs (*F*_1, 25,807_ = 974.02, *P* < 0.00001; *R*^2^ = 0.35 ± 0.024, *F*_2_ = 0.53, MSE = 2.23, RMSE = 1.50). The most relevant risk factors were: country (*β* = 1.46, *P* < 0.0001), physical activity (*β* = −1.13, *P* < 0.0001), mental health symptoms (*β* = 0.69, *P* < 0.0001), falls (*β* = 0.36, *P* < 0.0001), isolation (*β* = 0.3, *P* < 0.0001), SES (*β* = 0.27, *P* < 0.0001), heart disease (*β* = 0.27, *P* < 0.0001), diabetes (*β* = 0.22, *P* < 0.01), sex (*β* = 0.16, *P* < 0.0001), hypertension (*β* = 0.11, *P* < 0.0001), alcohol consumption (*β* = 0.11, *P* < 0.0001), age (*β* = −0.09, *P* < 0.0001), education (*β* = −0.08, *P* < 0.001), smoking status (*β* = −0.07, *P* < 0.001) and SES (*β* = 0.04, *P* < 0.0001).

The model also achieved significance in predicting functional ability for LA-HICs (*F*_1, 2,340_ = 44.85, *P* < 0.00001; *R*^2^ = 0.22 ± 0.061, *F*_2_ = 0.29, MSE = 3.13, RMSE = 1.77) (Fig. 2b). The most relevant risk factors were physical activity (lifestyle, *β* = 0.87, *P* < 0.0001), mental health symptoms (*β* = 0.55, *P* < 0.0001), falls (*β* = 0.52, *P* < 0.0001), country (*β* = 0.49, *P* < 0.0001), heart disease (*β* = 0.37, *P* < 0.0001), diabetes (*β* = 0.33, *P* < 0.01), SES (*β* = 0.27, *P* < 0.0001), isolation (*β* = 0.24, *P* < 0.05), hypertension (*β* = 0.18, *P* < 0.05), alcohol consumption (*β* = 0.15, *P* < 0.001) and age (*β* = −0.08, *P* < 0.0001).

When comparing the weight of each risk factor of functionality in LA-LMICs and LA-HICs using effect sizes (*F*_2_), all factors were statistically significant for LA-LMICs, while smoking status was not statistically significant for LA-HICs. The most relevant risk factors on LA-LMICs compared to HICs were physical activity, mental health symptoms, country, SES, isolation, falls, heart disease, diabetes and hypertension (Table 2 and Fig. 2b).

We also run a similar group of models to predict cognition and functional ability in each country. Across countries, and consistent with regional analysis, the more relevant risk factors of cognition were mental health symptoms, SDH, physical activity and education. In LA-LMICs (Colombia and Ecuador), age was also a significant risk factor of cognition although sex did not reach significant values. In LA-HICs (Uruguay and Chile), sex and age were significant risk factors of cognition. The more critical risk factors of functional ability in all LACs were a larger combination of mental health symptoms, SDH, lifestyle and cardiometabolic factors. While in LA-LMICs (Colombia and Ecuador), mental health symptoms, physical activity, SES and isolation had the highest scores to predict functional ability, in LA-HICs cardiometabolic factors, age and sex had a more relevant role (Extended Data Table 3).

### Longitudinal comparisons between Costa Rica and China

The most relevant risk factors of cognition and functional ability were investigated using longitudinal data taken from national surveys from Costa Rica; those results were compared with longitudinal data taken from a non-LA-UMIC (China). To this end, we derived the risk factors from the first wave and the outcomes (cognition and functional ability) from the last wave of the longitudinal survey assessments. We tested and compared the independent models for Costa Rica and China.

**Fig. 3:**
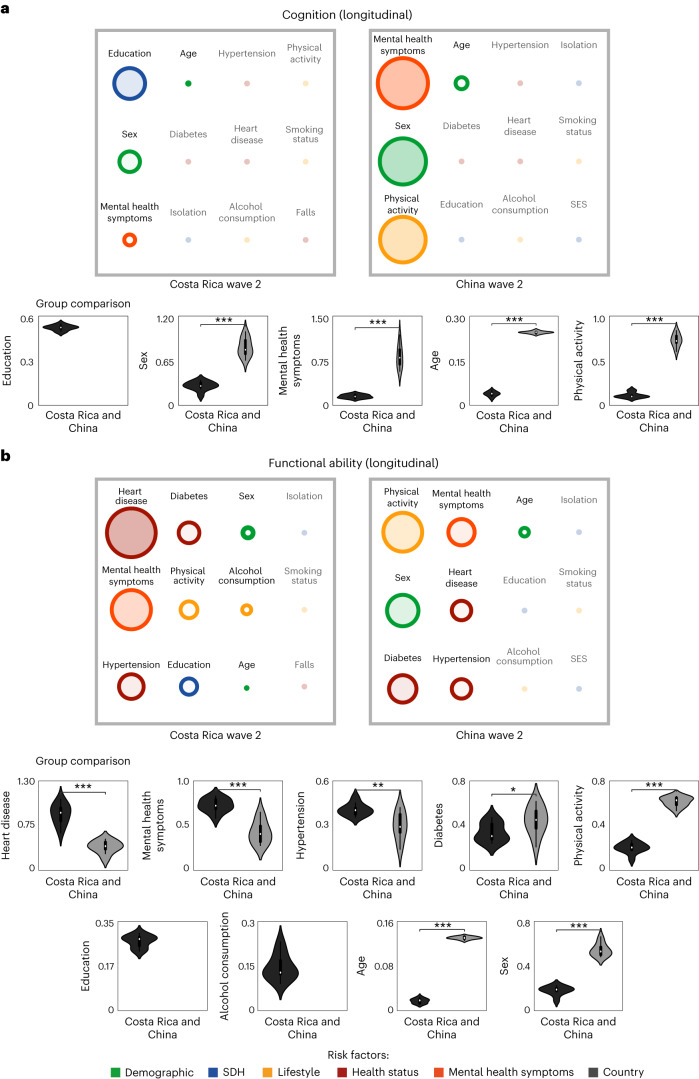
Longitudinal risk factors of cognition and functional ability in an LA-UMIC (Costa Rica) and a non-LA-UMIC (China). **a**,**b**, Longitudinal risk factors of cognition (**a**) and functional ability (**b**) were grouped into the following factors: demographics; SDH; lifestyle; health status (cardiometabolic factors and falls); mental health symptoms; and country. Features were ordered from most to least influential in the regression. The feature importance ranks in the regression model for cognition and functional ability are highlighted, accompanied by their statistical significance. Feature importance is represented by the radius of the circles and accentuated by the intensity of the color. The bottom parts of both panels show the countries’ comparison analyses (violin plots) used to test differences in the weight of significant risk factors (*β* estimates) of cognition and functional ability (*n* = 9,258). Ten iterations of the results were conducted to obtain ten *β* estimates for each risk factor, providing the minimum variance for performing group comparisons, which was analyzed with a two-sided Mann–Whitney *U*-test with Bonferroni correction. The specific values of the violin plots (minimum, maximum, center, 25th and 75th quartiles, inferior and superior whiskers) are provided in Extended Data Table 6 (for cognition) and Extended Data Table 7 (for functional ability). *P < 1.00 × 10^−2^ ≤5.00 × 10^−2^; **P < 1.00 × 10^−3^ ≤ 1.00 × 10^−2^; ***P < 1.00 × 10^−4^ ≤ 1.00 × 10^−3^.

**Extended Data Fig. 2:**
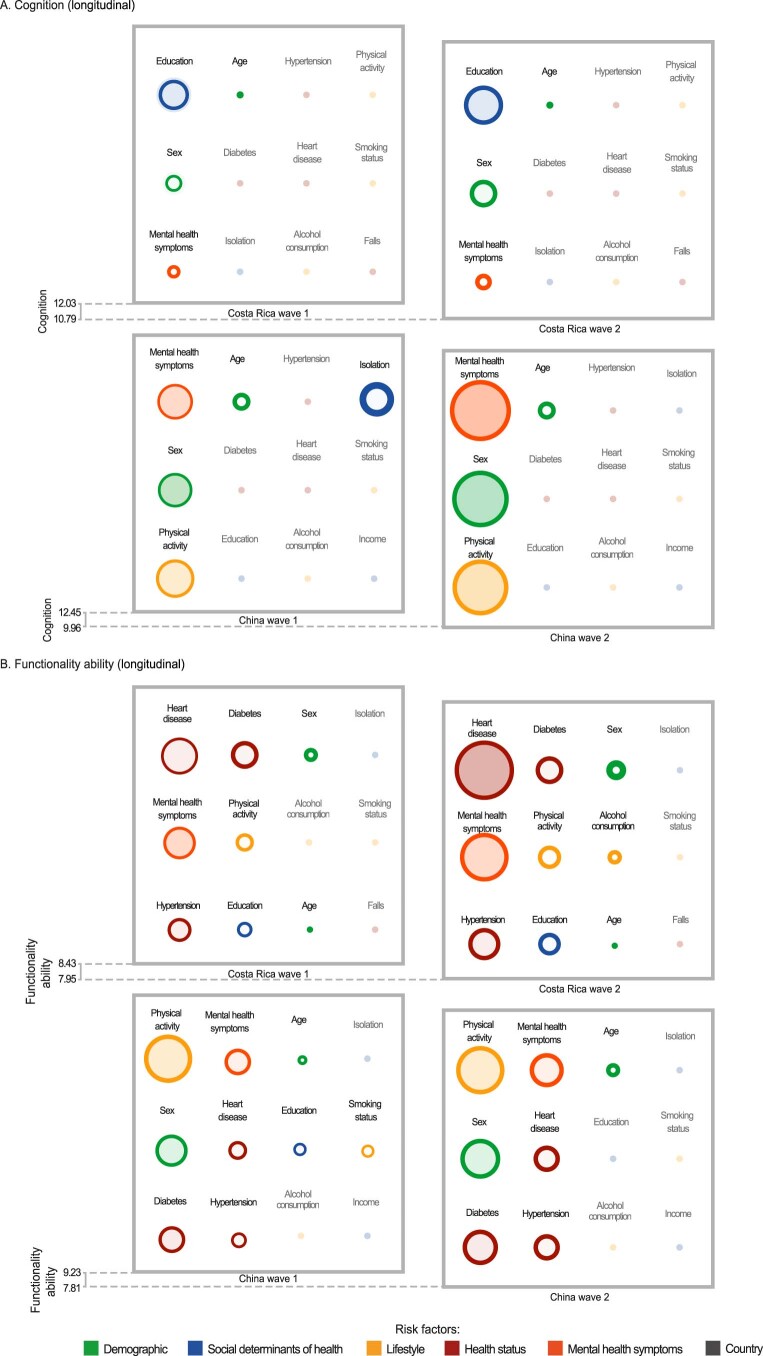
Longitudinal results of factors associated with cognition and functional ability. Longitudinal predictors of cognition and functional ability. Predictors of each wave of cognition (MMSE scores, panel A) and functional ability (Barthel scores, panel B) are shown for the LA-UMIC (Costa Rica) and the Asian UMIC (China). All panels demonstrated the significance of the predictors assessed in the initial wave for the outcomes evaluated in each wave (wave 1 and wave 2). The risk factors were grouped into fivefold categories: demographics, social determinants of health, lifestyle, health status, mental health symptoms, and country. The features were ordered from most to least influential in the regression. The feature importance is represented by the radius of the circles and accentuated by the intensity of the color.

We tested two independent models to assess the most relevant risk factors of cognition. The model predicting cognition was significant for Costa Rica (*F*_1, 5,694_ = 3.95, *P* < 0.00001; *R*^2^ = 0.14 ± 0.03, *F*_2_ = 0.15, MSE = 1.51 and RMSE = 1.22). The model assessing cognition for China also reached significant values (*F*_1, 3,112_ = 22.78, *P* < 0.00001; *R*^2^ = 0.21 ± 0.03, *F*_2_ = 0.23, MSE = 15.50 and RMSE = 3.95). For Costa Rica, the most relevant risk factors included education (*β* = 0.51, *P* < 0.00001), sex (*β* = 0.33, *P* < 0.00001), mental health symptoms (*β* = 0.18, *P* < 0.001) and age (*β* = 0.02, *P* < 0.0001). For China, the most relevant risk factors were mental health symptoms (*β* = −0.85, *P* < 0.00001), sex (*β* = 0.78, *P* < 0.00001), physical activity (*β* = 0.77, *P* < 0.00001) and age (*β* = 0.19, *P* < 0.00001). We ran an extra group of analyses to compare the weight of each significant risk factor of cognition between Costa Rica and China. Those analyses revealed that education was a stronger risk factor for Costa Rica than China. In contrast, age, sex and mental health symptoms were more relevant risk factors in China than in Costa Rica (Tables 3 and 4, Fig. 3a and Extended Data Fig. 2).

**Table 3:** Comparison of risk factors of cognition between Costa Rica and China in the longitudinal analyses using the Ridge regression models

Risk factors	Feature (taken in wave 1)	Costa Rica cognition (MMSE) of wave 2			China cognition (MMSE) of wave 2		
		*β* estimates	*t*	*P*	*β* estimates	*t*	*P*
Demographics	Intercept	12.01	7.53	NS	24.07	10.32	NS
	Age	0.024	8.61	<0.0001	0.19	80.10	<0.00001
	Sex	0.33	2.66	<0.01	0.78	2.66	<0.0001
SDH	Education	0.51	7.14	<0.00001	0.26	0.85	NS
	SES	0	0	NS	0.01	0.38	NS
	Isolation	0.19	0.95	NS	0.44	0.90	NS
Health status	Diabetes	0.12	0.85	NS	0.14	0.23	NS
	Hypertension	0.03	0.32	NS	0.16	0.56	NS
	Heart disease	0.20	0.65	NS	0.15	0.38	NS
	Falls	1.85	3.29	NS	0	0	NS
Mental health symptoms	Mental health symptoms	0.16	2.10	<0.05	0.85	2.19	<0.05
Lifestyle	Physical activity	0.03	0.30	NS	0.77	2.73	<0.01
	Alcohol consumption	0.07	0.77	NS	0.10	0.33	NS
	Smoking status	0.07	0.59	NS	0.13	0.42	NS

Ten iterations of results were conducted to obtain ten *β* estimates for each predictor, providing the minimum variance for group comparisons, which was analyzed with a two-sided Mann–Whitney *U*-test, with Bonferroni correction.

**Table 4:** Comparison of risk factors of functional ability between Costa Rica and China in the longitudinal analyses using the Ridge regression models

Risk factors	Feature (taken in wave 1)	Costa Rica functional ability (Barthel) of wave 2			China functional ability (Barthel) of wave 2		
		*β* estimates	*t*	*P*	*β* estimates	*t*	*P*
Demographics	Intercept	11.79	1.92	NS	16.12	2.01	NS
	Age	0.01	4.90	<0.00001	0.13	9.29	<0.00001
	Sex	0.17	1.09	<0.05	0.52	3.02	<0.0001
SDH	Education	0.27	3.01	<0.0001	0.15	0.82	NS
	SES	0	0	NS	0.08	0.45	NS
	Isolation	0.06	0.23	NS	0.13	0.46	NS
Health status	Diabetes	0.34	1.94	<0.0001	0.46	1.31	<0.01
	Hypertension	0.42	3.08	<0.00001	0.32	1.88	<0.0001
	Heart disease	0.84	2.16	<0.00001	0.32	1.36	<0.01
	Falls	0	0	NS	0	0	NS
Mental health symptoms	Mental health symptoms	0.67	3.99	<0.00001	0.44	1.91	<0.00001
Lifestyle	Physical activity	0.28	1.87	<0.0001	0.65	3.96	<0.00001
	Alcohol consumption	0.13	1.12	<0.05	0.17	1.00	NS
	Smoking status	0.04	0.32	NS	0.17	0.95	NS

Ten iterations of results were conducted to obtain ten *β* estimates for each predictor, providing the minimum variance for group comparisons, which was analyzed with a two-sided Mann–Whitney *U*-test, with Bonferroni correction.

We used an independent model to assess the most relevant risk factors of functional ability in each country. The model predicting functional ability for Costa Rica reached significant values (*F*_1, 5,694_ = 5.67, *P* < 0.00001; *R*^2^ = 0.14 ± 0.0222, *F*_2_ = 0.16, MSE = 2.08 and RMSE = 1.44). The model predicting functional ability for China also was significant (*F*_1, 3,563_ = 28.7, *P* < 0.00001; *R*^2^ = 0.24 ± 0.032, *F*_2_ = 0.31, MSE = 6.30 and RMSE = 2.51). The most relevant risk factors of functional ability for Costa Rica were heart disease (*β* = 0.84, *P* < 0.00001), mental health symptoms (*β* = 0.67, *P* < 0.00001), hypertension (*β* = 0.42, *P* < 0.00001), diabetes (*β* = 0.35, *P* < 0.00001), education (*β* = 0.27, *P* < 0.00001), physical activity (*β* = 0.27, *P* < 0.00001), sex (*β* = 0.17, *P* < 0.0001), alcohol consumption (*β* = 0.15, *P* < 0.0001) and age (*β* = 0.01, *P* < 0.00001). For China, the critical risk factors were physical activity (*β* = 0.065, *P* < 0.0001), sex (*β* = 0.52, *P* < 0.00001), diabetes (B = 0.46, *P* < 0.0001), mental health symptoms (*β* = 0.44, *P* < 0.0001), heart disease (*β* = 0.32, *P* < 0.0001), hypertension (*β* = 0.32, *P* < 0.0001) and age (*β* = 0.13, *P* < 0.00001; Tables 3 and 4, Fig. 3b and Extended Data Fig. 2).

We compared the countries’ significant risk factors of cognition and functional ability by using the *β* estimates of each significant risk factor (Fig. 3a,b, bottom). Those analyses showed higher predictive scores for education, hypertension, heart disease and mental health symptoms in Costa Rica than in China. Moreover, education and alcohol consumption were the only significant risk factors in Costa Rica. In contrast, age, sex and physical activity reached higher predictive values for China than for Costa Rica.

### Imputation and complementary analyses

We ran models with individuals who had complete values in all variables measured in all countries. However, we ran the same group of analyses using the imputation-by-means method to handle missing values from one variable for the Costa Rica dataset in models of prediction of cognition and functional ability. The imputation method allows for increasing the number of observations when variables are assumed to exhibit a reduced number of outlier values while favoring data interpretation^26^. We implemented this procedure to maintain the same number of variables to compare models between Costa Rica and China. The results were consistent after running regression models for both cognition and functional ability with the imputation method (Extended Data Table 4).

We also performed complementary analyses to determine the independent associations between specific risk factors (sex, age, mental health status, lifestyle, health status, including cardiometabolic factors, and SDH) and healthy aging outcomes. These analyses highlighted the significant effects of each risk factor when examined independently (Extended Data Table 5).

## Discussion

The present study aimed to assess the most relevant risk factors of healthy aging (cognition and functional ability) across different LAC countries. In line with our hypothesis, the results revealed a heterogeneous and distributed set of social and health disparity-related risk factors of cognition (mental health symptoms, SDH, education and physical activity) and functional ability (mental health symptoms, SDH, education, physical activity and cardiometabolic factors) across LAC countries. Such heterogeneous sets of risk factors were more accentuated in LA-LMICs compared to LA-HICs. The longitudinal study yielded similar results by comparing an LA-UMIC (Costa Rica) and a non-LA-UMIC (China) as social and health disparity factors reached a higher predictive role in Costa Rica than in China, and a more complex group of features determined functional ability in Costa Rica, in contrast to the classical demographic risk factors in China. Across all analyses, disparity-related social and health risk factors were more significantly associated with healthy aging than classical factors such as age and sex in LAC countries.

Regarding cognition, SES is a relevant risk factor^1,27^ having a crucial role in this study. Cognition and low SES have been linked to specific SDH, such as social exclusion, isolation and reduced social interactions^28,29^, which are more prevalent in LAC countries^30^, especially in older adults^31^. Mental health symptoms were identified as a top risk factor, probably due to their increased prevalence across LAC countries compared to other regions^5,19,32–34^. Cardiometabolic factors did not reach significant scores as reported previously^18^. One possible interpretation could be the limited ability of the scale used to measure cognition, the Mini-Mental State Examination (MMSE), to identify executive functioning and reduced capacity to capture the early stages of cognitive decline^35^. However, this null association could be better explained by complex interactions between risk factors in our models. This interpretation was supported by complementary analyses demonstrating the significant effects of cardiometabolic factors when analyzed separately. Future studies should systematically assess how combining different potential risk factors^15^ could modify the model’s predictive role. Regardless, our results highlight a heterogeneous combination of risk factors impacting cognition^36^ associated with regional inequity and specificity.

Regarding functionality, our results confirm the role of physical activity in functional ability^37^, probably through reducing noncommunicable diseases^37^. Mental health symptoms, which are highly prevalent in LAC countries^5,19,32–34^, may reduce autonomy and motivation for daily activities^38^. Falls also impacted functional ability^39^, which may be exacerbated in LAC countries by the barriers to accessing health and social support^40^. Although cardiometabolic factors are associated with functional ability^41,42^, this association was less pronounced than with other factors. Social isolation and SES^41,43^, usually accentuated in LAC countries^41^, were also relevant risk factors. Thus, a heterogeneous combination of risk factors related to regional disparities was associated with cognition and functional ability.

Classical risk factors associated with cognition and functional ability, such as age and sex, were less accentuated than those related to social and health disparities, unlike in other regions^1,10–14,42^. Although these factors were significant when analyzed independently, their effects diminished or disappeared when combined with other risk factors. In LAC countries, there is an increased presence of SDH^17,31^, an augmented prevalence of cardiometabolic factors^18^ and mental health symptoms^5,19,32^, and barriers to healthy lifestyles^20,21^. The combination of these disparity-related risk factors in LAC countries could attenuate the impacts of sex and age as risk factors of healthy aging. In contrast, classical risk factors may have a more substantial role in healthy aging in Europe and the United States. In those regions, there is also a more substantial control of modifiable social and health disparities, which would help diminish their effects and explain the contrasting pattern. Similarly, social and health disparities were stronger risk factors in LA-LMICs than in LA-HICs, which is consistent with the larger inequalities in the former^9^. Also, differences between Costa Rica and China confirmed this pattern despite their similar socioeconomic development (Extended Data Tables 6 and 7). In summary, heterogeneous and disparity-related factors were critically associated with cognition and functional ability in LAC countries, especially in LA-LMICs, and presented stronger influences than other classic factors such as age and sex.

Our approach based on machine learning methods can address multicollinearity and high-dimensional data^44^ and incorporate sample validation processes, thereby providing a more reliable assessment of the model’s performance on unseen data than classical statistical models^44^. Our modeling approach can handle complex interactions between risk factors and outcomes more effectively than standard regressions and other traditional statistical methods to assess associations between variables^45^. Similarly, it can identify the top predictors without assuming a priori theoretical rankings, which are usually required when classical statistical methods are applied^44^ (Extended Data Table 8).

There are limitations to our study. First, as in previous reports of SDH and cardiometabolic factors^46^, data collection is based on self-reports and could be prone to bias. Our study combined self-reported data and standardized objective measures to diminish such potential bias. Still, future assessments with objective measures would be needed to confirm our results. Second, the large population might have impacted statistical significance and reduced prediction accuracy in machine learning algorithms^47^. We handled these concerns using a sample size robust enough for each statistical comparison and effect size estimation; combined statistical indexes confirmed the machine learning accuracy obtained. Our study only used MMSE and the Barthel index as the primary measures of cognitive and functional outcomes in the context of healthy aging, which may not capture all aspects of healthy aging. Finally, some potential risk factors of aging, such as mental health symptoms, were assessed using a single self-reported question. Although previous studies have assessed mental health symptoms using similar procedures^32,48^, future studies should consider using more comprehensive tools to evaluate healthy aging outcomes, mental health symptoms and other relevant factors.

While several global organizations, including the World Health Organization^22,49^ and the Alzheimer’s Association^1,24^, have called for the improvement of public health actions regarding healthy aging in LAC countries, initiatives are still limited, generic and poorly targeted to this region. The result of our study encourages public health leaders to consider the complex interactions of multiple disparity-related factors^4,15^, including individual health-related markers and SDH. By understanding region-specific risks, policymakers can develop tailored prevention responses. Our study invites public health actions to prioritize programs to address multimodal disparities and promote mental health across the life span, mainly in older ages. Additionally, our study calls for developing national plans to increase population education and promote public resources^50^ to favor social networks and healthy lifestyles, particularly stimulating physical activity. This comprehensive approach can be articulated with public health programs focused on reducing the risks associated with noncommunicable diseases, which are highly prevalent in LAC countries. Our results also invite us to develop social and health plans to handle several aging risks simultaneously rather than reacting to one factor at a time. This multifaceted strategy provides a robust foundation for informing policies with synergistic effects across multiple conditions, optimizing resource allocation in public health and fostering healthier societies.

In conclusion, our results highlight a differential, region-specific and granular set of risk factors for cognition and functional ability in LACs. The findings reveal risk factors according to region and country, strongly influenced by the greater inequalities that exist in LAC populations. Income status and SDH, specific disease burden, health practices and ancestry-related factors may defy standard models of healthy brain aging, suggesting that the one-size-fits-all approach does not necessarily work. Tailored models should better inform local and regional public health initiatives grounded in more diverse, region-specific approaches.

## Methods

### Cross-sectional analyses of participants

Participants (*n* = 31,680; female = 16,074; mean age = 70.81 years, s.d. = 8.22) were recruited from the national surveys on health, well-being and aging performed in four LAC countries: Chile (2016, *n* = 1,301; females = 689; mean age = 71.83 years, s.d. = 8.29)^51^; Uruguay (2011, *n* = 1,450; females = 798; mean age = 70.75 years, s.d. = 7.40)^51^; Ecuador (2012, *n* = 5,235; females = 1,530; mean age = 70.09 years, s.d. = 7.82)^52^; and Colombia (2015, *n* = 23,694; females = 1,357; mean age = 70.79 years, s.d. = 8.26)^53^. The interviews from all national surveys were performed face to face and participants were selected following a probabilistic, clustered, stratified and multistage design in each country. We only included individuals with no diagnosis of dementia, as assessed during an initial screening (Fig. 1a–e). The databases of national surveys from all countries are open and were obtained according to the established procedures for each country.

### Longitudinal analyses of participants

We analyzed data from the national aging study of an LA-UMIC: Costa Rica (Costa Rican Study on Longevity and Healthy Aging (CRELES), *n* = 5,694; ref. ^54^). This survey includes two waves of information on the risk factors of aging (wave 1 = 2012, wave 2 = 2015–2016). Longitudinal information from a non-LA-UMIC in China (Chinese Longitudinal Healthy Longevity Survey Series, *n* = 3,564; ref. ^55^) was used as control information. This survey includes two waves of information (wave 1 = 2011, wave 2 = 2014–2015). In this longitudinal approach, we assessed the role of factors taken in the first wave in predicting the scores of measures of cognition and functional ability of wave 2. The final population from both the Costa Rica and China datasets totaled 9,258 participants (Fig. 1b).

The interviews from all national surveys were performed face to face and participants were selected following a probabilistic, clustered, stratified, multistage design in each country. Information collected in all countries, in the context of national aging surveys, was representative of each population (Colombia, Ecuador^52^, Uruguay^51^, Chile^51^, Costa Rica^54^ and China^55^). The instruments, harmonization and computational approaches of this study followed the same parameters as described in the cross-sectional analyses.

### Instruments

#### Risk factors

The final datasets from the surveys in all LAC countries consisted of five harmonized risk factors (demographics, SDH, health, lifestyle and mental health symptoms).

##### Demographics

Demographics included age (years), sex (women and men only) and years of education attained. No questions were asked regarding gender identity aspects in any country.

##### SDH

To assess this risk factor, we included information previously associated with brain health^50^, including measures of isolation (whether the participant lives alone or not), SES (which was measured with a composite index that included income and housing conditions) and educational level (which has three levels: low (elementary school and middle school), medium (high school) and high (bachelor’s and postgraduate degrees).

##### Health status

This included health factors previously associated with brain health such as cardiometabolic factors (hypertension, diabetes and cardiovascular risks) and falls.

##### Lifestyle

This included smoking behavior, alcohol consumption, and physical activity.

##### Mental health symptoms

We collected information on the presence of depression, anxiety and other mental health symptoms. Those symptoms were categorized as binomial variables.

#### Outcome variables

##### Cognition

Cognition was assessed in national surveys from LAC countries with the shortened version of the Folstein MMSE. Following previous procedures^56,57^, we used the abbreviated MMSE, which has 19 questions and items. A cutoff of 13 points was used in each survey to determine if the participant had cognitive decline^57^.

##### Functional ability

The Barthel index^58^ assesses difficulties in ADLs, including basic activities of daily life such as self-maintenance skills (dressing, bathing, grooming, toilet use, and bowel and bladder continence) and mobility skills^56^. We analyzed information from a group of activities collected via participants’ interviews and harmonized across countries referring to daily living functioning. The scores were built in each database by computing a ratio of positive responses according to previous studies^58^. For a further description of specific questions to assess the risk factors and the healthy aging outcomes used in each country, see the Supplementary Information.

### Data harmonization of variables across countries

We ran harmonization procedures^59^ for some variables because the answer options of some measures were different between countries. Similarly, missing values were observed in some datasets. All variables used in the analyses of this study had complete values in at least 80% of cases. Thus, we collected 28,109 participants with complete values in the variables of the analyses to assess risk factors of cognition and a population of 27,991 participants for functional ability (Fig. 1a–e).

#### Harmonization procedures of variables across countries in the cross-sectional analyses

##### MMSE

The test contained the following questions: (1) participants should provide the date. This question scores one point for each of the following correct answers: day of the week, day of the month, month and year; (2) the interviewer names three objects and the participants must remember them. This question scores one point for each object recalled; (3) the interviewer gives the individual a piece of paper to perform three actions. Performing each action correctly scores one point; the actions are: taking a piece of paper with the right hand, folding the paper in half with both hands and placing the paper on the lap; (4) the participant remembers the name of the previous three objects again. This question is scored the same as question 2; (5) participants copy a drawing. One point is scored if the drawing is done correctly. The final scale is achieved using standardized scores of the abbreviated MMSE except a question about remembering a sequence of five numbers, which scored five points. This question was removed because it was evaluated in different ways across the databases and no complete information was recruited to harmonize the scores across sites.

##### SDH harmonization

Educational level was set to three levels: low (elementary and middle school); middle (high school); and high (bachelor’s and postgraduate degrees). SES was calculated on the proportion of available housing resources and services.

##### Lifestyle harmonization

Alcohol consumption was set to four levels: never (participant never consumes alcohol); normal (participant consumes alcohol less than one day per week); overdrinking (participant consumes alcohol two to six times per week); severe (participant consumes alcohol daily). All other variables were dichotomous (yes/no), except for age, which was collected by all countries as a discrete variable. Non-common features were dropped.

#### Harmonization procedures of variables across countries in the longitudinal analyses

The MMSE information was computed on 13 points in Costa Rica and China because the drawing test was not conducted in Costa Rica. We also ran imputation by means to impute the missing values of one feature (falls) in the Costa Rica dataset. For imputation, we used the mean scores in the aforementioned variables from other HICs (Chile and Uruguay). To control for the effects of imputation, additional analyses without imputation were also performed (Extended Data Table 4).

### Statistical analysis

No statistical method was used to predetermine sample size because we used the complete database of the national aging surveys from different LAC countries. All datasets from the national aging surveys from LAC countries in cross-sectional and longitudinal analyses included representative samples from each country.

#### Cross-validation for hyperparameter tuning

First, we conducted the best search for the most appropriate set of hyperparameters using cross-validation to obtain the best possible generalization results^60,61^. For each model, we implemented a Bayesian optimization approach for hyperparameter tuning, with cross-validation = 3 and ten iterations, on 75% of the data (training dataset), and evaluated the results on a validation dataset (25% of the data). Then, we randomly divided the data on a new training sample (75%) and testing sets (25%) into *k* = 10 folds; each subset was used for training *k* − 1 times and validation, using the best hyperparameters obtained from the Bayesian optimization in the previous step. Finally, we obtained the margins of errors, the *β* estimates and *t*-tests by assessing the mean of *k* = 10 iterations. This facilitated the identification of optimal regularization strength and additional hyperparameters for the ridge regression model.

#### Solver selection

We optimized the regularization strength, the maximum number of iterations and the solver. We followed scikit-learn’s implementation for ridge, which allows testing and comparing different solvers, including: (1) auto: it chooses the solver automatically based on the data. This solver reached 1,000 maximum iterations and an alpha of 0.0001; (2) singular value decomposition involving features vectors^62^. This approach had 10,000 maximum iterations and an alpha of 0.001; (3) Cholesky, a standard linear function that allows obtaining a closed-form solution (Extended Data Table 8). The maximum number of iterations with this approach was 100,000 and the alpha was 0.01; (4) sqr, a dedicated regularized least-square routine, and sag, a stochastic average gradient descent^63^, which uses a maximum number of iterations of 1,000,000; and (5) sparse_cg, a conjugate gradient solver involving sag and saga that follows the stochastic average gradient descent process and an optimized version of this approach, respectively^63^ (Extended Data Table 2).

Moreover, we used different regularization and solver selection processes for elastic net and LASSO. For elastic net, we used two solvers including (1) cyclic, which repeats features sequentially by default with a maximum number of iterations of 1,000 and an alpha of 0.0001; and (2) random, which updates a random coefficient in every iteration, with a maximum number of iterations of 10,000 and an alpha of 0.001. Moreover, different L1 ratios (ranging from 0.5 to 0.9) were used, and the iterations and alpha were fitted according to this ratio. For LASSO, we implemented two solvers, including: (1) cyclic with a maximum number of iterations of 1,000 and an alpha of 0.0001; and (2) random with a maximum number of iterations of 10,000 and an alpha of 0.001 (Extended Data Tables 2 and 8). The score we used for optimization was the square loss, which is used in ridge regressions as default^64^. This method imposes greater penalties on larger errors compared to smaller ones^64^.

The closed-form solution for ridge regression, which includes a regularization parameter *ɑ* to control the amount of shrinkage, can be expressed as follows:

$$\begin{array}{c}{{\rm{min }}}_{{{w}}}||{{Xw}}-{{y}}|{|}^{(2)}+{{a}}||{{w}}|{|}^{(2)}\\ {\min }_{[{{w}}]}||{{X}}\,{{w}}-{{y}}|{|}_{2}^{2}+\alpha ||{{w}}|{|}_{2}^{2}\end{array}$$

where *w* represents the coefficients of the regression model; *X* is the design matrix, where each row represents an observation and each column represents a risk factor variable; *y* is the target variable; and *ɑ* is the regularization parameter (also known as the ridge parameter), which controls the amount of shrinkage: the larger the value of *ɑ*, the greater the amount of shrinkage.

The parameters of *ɑ* used for our analyses are provided in Extended Data Table 8.

#### Ridge regression method

We assessed the correlation and collinearity between risk factors (as revealed by the variance inflation factor, scores above 5). Those analyses showed multicollinearity (Fig. 1g and Extended Data Table 1) and correlations among variables (Extended Data Fig. 1). Thus, we chose ridge regression models to assess the more relevant risk factors of healthy aging. This method helps to (1) handle the risk factors’ multicollinearity and correlations; (2) reduce overfitting because it introduces a regularization term to penalize large coefficients, thus improving the model’s generalization^64^; (3) improve model stability because it shrinks the coefficients toward zero, effectively reducing the impact of noise or irrelevant features on the model’s predictions^65^; (4) improves model interpretability because it shrinks the coefficients, which helps interpret the importance of different risk factor variables selected based on previous theoretical accounts^66^; and (5) addresses variance and interactions because it is a recommended process to tackle regression challenges in the presence of multidimensionality and complex interactions between risk factors^67^.

The ridge regression model using cross-sectional data can be expressed as:

$$\min (\,\beta )||Y-X\beta |{|}^{2}+\lambda ||\beta |{|}^{2}$$

where *Y* is the outcome variable, *X* is the matrix of the risk factor variables, *β* is the vector of the coefficients and *λ* is the regularization strength.

The ridge regression model for longitudinal data can be expressed as:

$$\hat{y}=X(\,\beta )+\varepsilon ,\,\mathrm {subject}\,\mathrm {to}||\beta |{|}_{2}^{2}\le t$$

where *ŷ* represents the predicted outcome, *X* is the matrix of the risk factors, *β* is the vector of the coefficients, *ε* is the error term, ${{||}\beta ||}_{2}^{2}$ is the L2-norm of *β* (sum of squared coefficients) and *t* is the regularization parameter.

#### Multimethod analyses

To confirm the robustness of our approach, we implemented multimethod confirmatory analyses (Fig. 1f,g,k). This comparative assessment involved examining the outcomes of ridge regression in conjunction with linear regression, elastic net and LASSO techniques. Linear regression was used to fit a predictive model to the observed data. It calculates the strength of the relationship between risk factors and outcome variables and helps to determine whether some explanatory variables may have no linear relationship with the outcomes. Elastic net combines the advantages of ridge and LASSO regression, incorporating both L1 and L2 regularization^68^. Furthermore, it balances the benefits of both techniques, providing a compromise between the sparsity of LASSO and the stability of ridge regression. This can result in better prediction accuracy and improved model interpretability^68^. LASSO regressions encourage sparsity in the model by driving some coefficients to zero, leading to more straightforward and more interpretable models, and allowing for automatic feature selection to deal with many risk factors^69^.

#### Goodness-of-fit and weight of risk factor parameters

We used different parameters to assess the goodness of fit in the studies, including (1) *F*-statistic (*F*) for the regression coefficients of the models; (2) *R*^2^, a measure of how close the data points correspond to the fitted line and as the coefficient of determination for the regression models; (3) Cohen’s *F*_2_ to assess the effect sizes of the regression models and risk factors; (4) MSE, an estimator of the average of the squared difference between estimated and actual values and to assess the goodness of fit of the regression models; (5) the RMSE as a measure of the standard deviation of the residuals (prediction errors) and to assess the distance between the regression line data points; and (5) *β* estimates to assess the weight of a factor in a regression model. Different linear regression models were used to identify the best goodness-of-fit parameters across the analyses. The multimethod procedure confirmed the consistency of the results and suggested that linear approaches were the best choice. Other additional nonlinear methods are out of the scope of this study.

#### Cross-sectional analyses

We ran two independent ridge regression models to assess the risk factors of healthy aging: one model was run to assess the risk factors of cognition (MMSE scores as the outcome), and the other was implemented to assess functional ability (Barthel scores as the outcome) risk factors across all LAC countries. Risk factors including demographics, SDH, health status, lifestyle and mental health symptoms were included as risk factors in each model. Ridge regression models were first run for cognition and functionality across all LAC countries (Colombia, Ecuador, Chile and Uruguay). Second, the regression models were run to group LAC countries according to their income level: HICs (Chile and Uruguay) and LMICs (Colombia and Ecuador). Third, independent regression models were run for each country. We evaluated the regression models by reporting *R*², 99% of confidence intervals, feature significance and *β* estimates. Each model considers the *F* value and the *F*_2_ values to assess the effect sizes (*F*_2_ ≥ 0.02, *F*_2_ ≥ 0.15 and *F*_2_ ≥ 0.35 representing small, medium and large effect sizes, respectively^70^).

#### Longitudinal analyses

The longitudinal evolution of outcomes (cognition and functional ability in two different moments for Costa Rica: wave 1 (2012) and wave 2 (2016); two for China: wave 1 (2011) and wave 2 (2014)). We computed the most relevant risk factors of cognition and functional ability in the last wave for Costa Rica and China using the risk factors of wave 1. Independent ridge regression models comprised the best hyperparameters using the scheme described in the cross-sectional analyses. All models and statistical analyses were run using Python v.3.9.13.

### Ethics and inclusion statement

This work involved a collaboration between scientists in multiple countries including Argentina, Chile, Colombia, Ireland, Peru and the United States. Contributors from all sites are included as coauthors or in acknowledgements according to their contributions. Researchers residing in Latin American countries have been involved in study design, study implementation, methodological procedure, and writing and reviewing processes. The current research is locally relevant due to the high prevalence of cognitive decline in LAC countries. Roles and responsibilities were agreed among collaborators ahead of the research. Local ethics committees approved all research involving human participants. To prevent any stigmatization, all identifying information has been removed to preserve the privacy of individuals. Each country included in this study have retained ownership of all human material shared for research purposes.

We endorse the Nature Portfolio guidance on LMIC authorship and inclusion. Authorship was based on the intellectual contribution, commitment and involvement of each researcher in this study. We included authors born in LMICs and other underrepresented countries in this study. This study holds local relevance for each investigated country by presenting disaggregated findings, thereby offering country-specific risk factors of healthy aging. The selection of variables was informed by previous research and in accordance with established guidelines for global aging studies.

### Ethical approval

All methods were carried out in accordance with relevant guidelines and regulations provided by the Declaration of Helsinki (2013). Data for all countries including LMICs were collected via in-person interviews implemented in the context of national aging surveys taken in each country.

In each country, participants gave informed consent, which was approved by the respective ethics committees. Data collection and analysis posed no risks concerning stigmatization, incrimination, discrimination, animal welfare, environmental, health, safety, security or personal concerns. No transfer of biological materials, cultural artifacts or traditional knowledge occurred. The authors reviewed pertinent studies from all seven countries while preparing the manuscript. The Pontificia Universidad Javeriana’s ethical institutional committee in Bogotá, Colombia, approved all experimental protocols used in the analyses of this study (no. FM-773-2021).

### Reporting summary

Further information on research design is available in the Nature Portfolio Reporting Summary linked to this article.

## Online content

Any methods, additional references, Nature Portfolio reporting summaries, source data, extended data, supplementary information, acknowledgements, peer review information; details of author contributions and competing interests; and statements of data and code availability are available at 10.1038/s41591-023-02495-1.

## Supplementary information

Supplementary Information Supplementary Instruments. Description of predictors used in each country in the cross-sectional and longitudinal analyses.

Reporting Summary

## Figures and Tables

**Extended Data Table 1:**
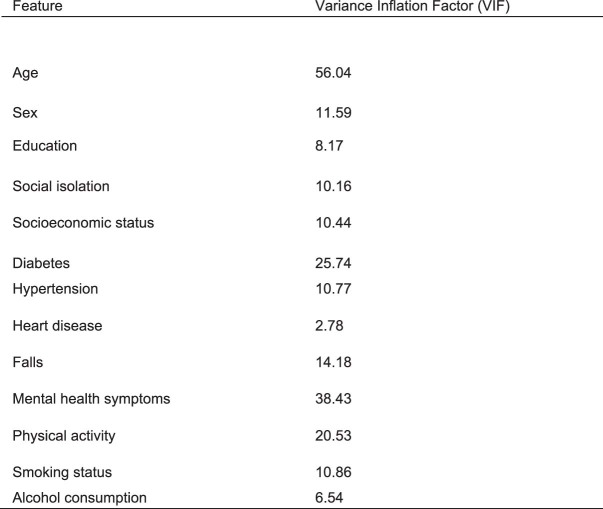
Collinearity test between factors associated with healthy aging

**Extended Data Table 2:**
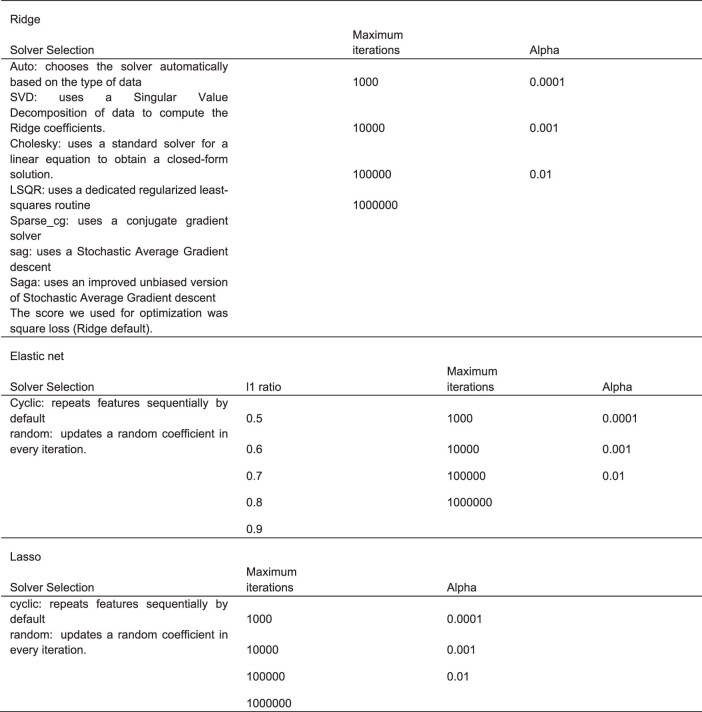
Ridge and elastic net regularization parameters in models used to assess factors associated with healthy aging

**Extended Data Table 3:**
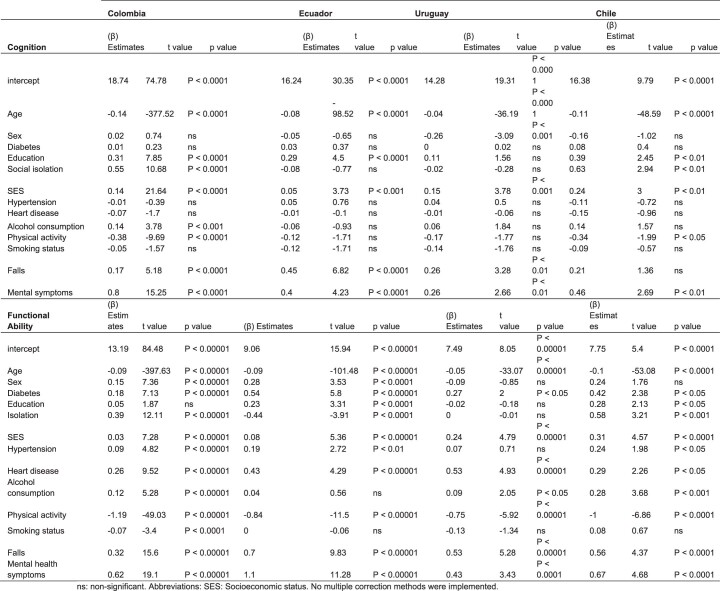
Predictors of healthy aging outcomes in each LAC. Ridge regression models of factors associated with healthy aging in each LAC

**Extended Data Table 4:**
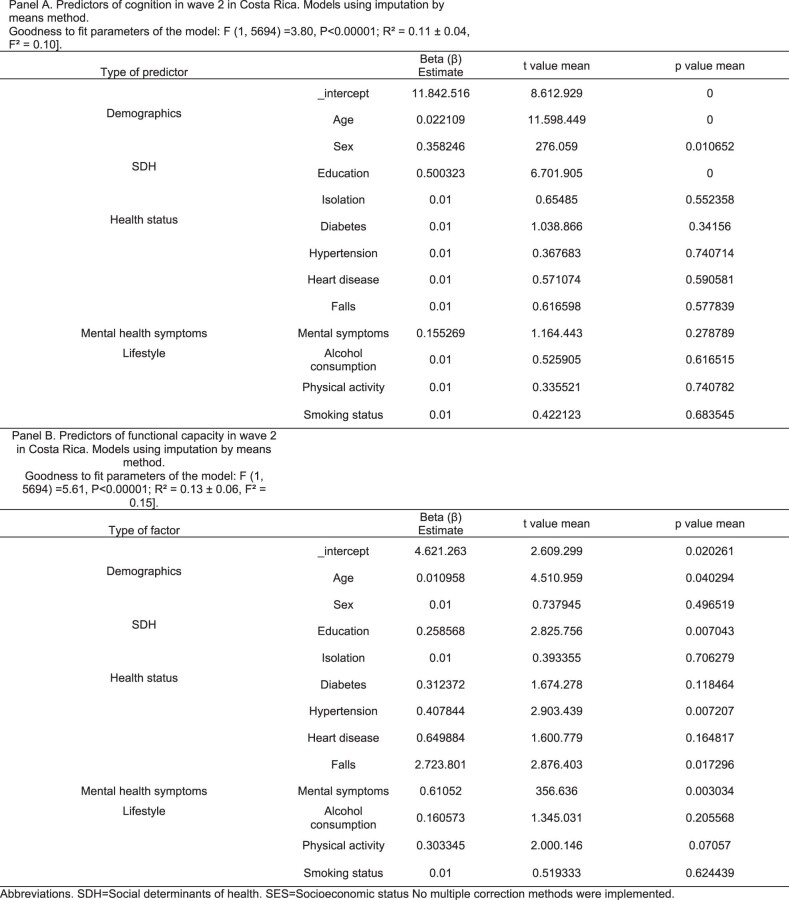
Ridge regression models of risk factors of healthy aging in longitudinal analyses after implementing imputation methods

**Extended Data Table 5:**
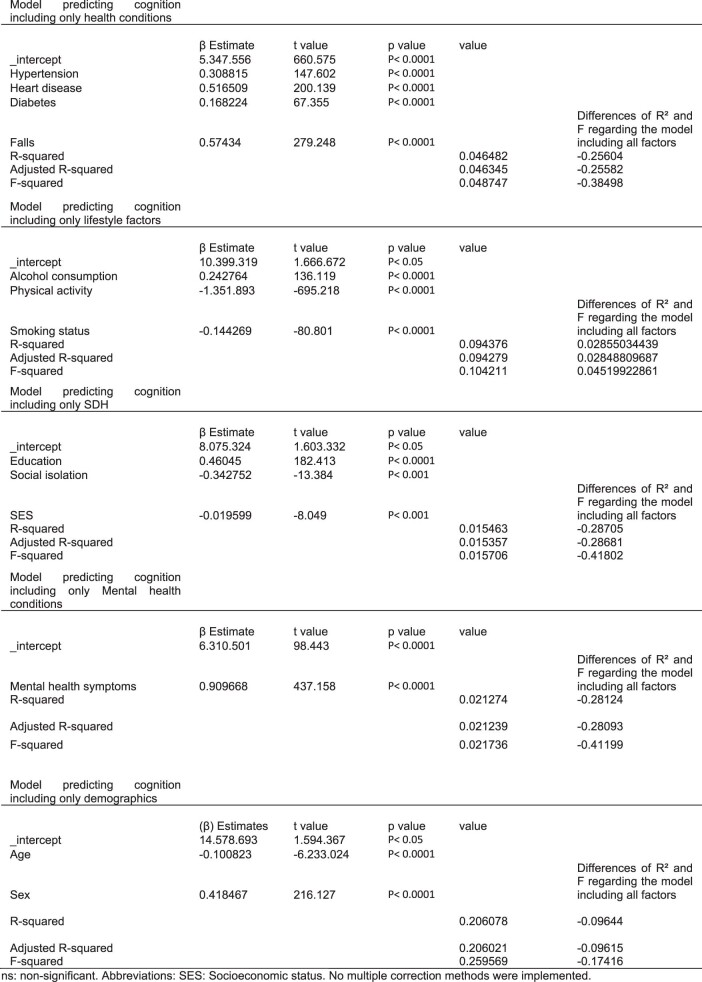
Ridge regression models of individual impact of each type of risk factor on healthy aging

**Extended Data Table 6:**
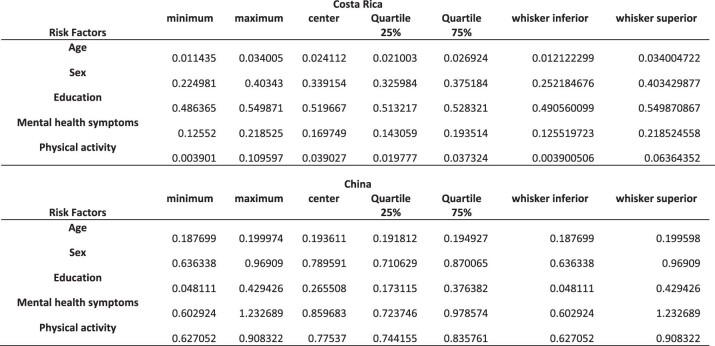
Group comparisons of the magnitude of risk factors of cognition between Costa Rica and China

**Extended Data Table 7:**
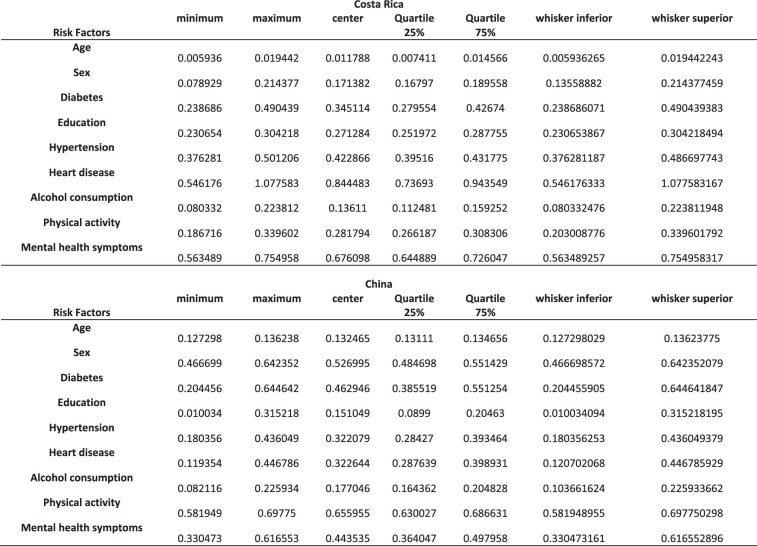
Comparisons of the magnitude of risk factors of functional ability between Costa Rica and China

**Extended Data Table 8:**
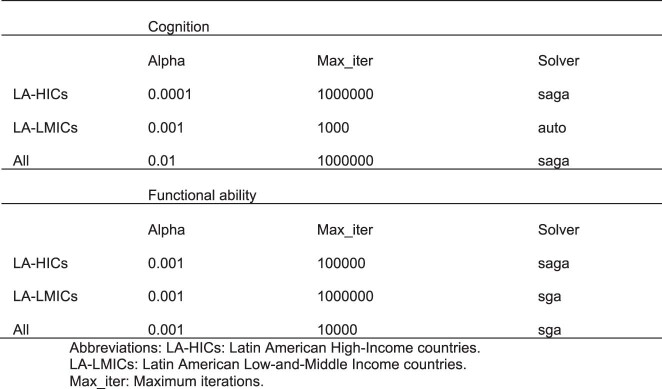
Closed-form solution for ridge regressions

## Data Availability

Data included in this study in the cross-sectional and longitudinal analyses were collected in the context of the national aging surveys of five LAC countries (Ecuador, Colombia, Chile, Uruguay and Costa Rica) and from China. Data from the national aging survey from Ecuador included in the study are publicly available and can be accessed after providing researchers information at https://www.ecuadorencifras.gob.ec/encuesta-de-salud-bienestar-del-adulto-mayor/. Data from the national aging survey of Colombia (National Study of Health, Well-being, and Aging (SABE Colombia) 2015) can be accessed after filling a registration form on the Web page of the Ministry of Health and Social Protection (el Ministerio de Salud y de Protección Social) in Colombia. This procedure lasts around 10 days and can be made at this https://www.datos.gov.co. Data from Chile and Uruguay included in this study were part of the SABE-Survey on Health, Well-being, and Aging in Latin America and the Caribbean, 2000 (Inter-university Consortium for Political and Social Research 3546). Data from this study are publicly available after providing researcher information at https://www.icpsr.umich.edu/web/NACDA/studies/3546/versions/V1. Data from Costa Rica included in this study were taken from CRELES. Data from this study are publicly available after providing researcher information at http://creles-download.demog.berkeley.edu/CRdata.pl. Data from the national aging survey of China (China Health and Retirement Longitudinal Study) can be accessed after registration at https://charls.pku.edu.cn/en/. Access to the raw data from all databases described in this section lasts around 10 days. All authors had access to the raw data. All software used in this study and its versions are specified on the conda_env.yml file in the GitHub repository.

## Code availability

All code for the data analysis associated with the manuscript is available for download from GitHub at https://github.com/AI-BrainLat-team/Heterogeneous-risk-factors-LAC.
