# Supplementary material for: Factors associated with healthy aging in Latin American populations
Source: Nat Med. 2023 Aug 10;29(9):2248–58. doi: 10.1038/s41591-023-02495-1 (PMC10504086; doi:10.1038/s41591-023-02495-1)
Supplement: Supplementary file 1 — Supplementary Instruments. Description of predictors used in each country in the cross-sectional and longitudinal analyses. [file 41591_2023_2495_MOESM1_ESM.pdf]

nature medicine

Article https://doi.org/10.1038/s41591-023-02495-1

Factors associated with healthy aging in Latin American populations

In the format provided by the authors and unedited

Description of healthy aging measures and predictors used in cross-sectional analyses

Healthy aging measures

Cognition

This domain was assessed using the Minimental State Examination (MMSE)[94].

Below we described the questions used in each country referring to cognition.

Chile/Uruguay/Ecuador/Colombia: This metric was computed on 14 points by adding the correct answers or actions of a 5-question test. The test contained the following questions: Question 1. Subjects tell the date. This question scored 1 point for each of the following correct answers: the day of the week, the day of the month, the month and year. Question 2. The interviewer names 3 objects and the subjects must remember them. This question scored 1 point for each object recalled. Question 3. The interviewer gives the subject a piece of paper to perform 3 actions. Performing each action correctly scored 1 point, the actions were: taking a piece of paper with the right hand, folding the paper in half with both hands and placing the paper on her/his lap. Question 4. Subjects remember the name of the previous 3 objects again. This question was scored the same as question 2. Question 5. Subjects copy a drawing, in this case, 1 point was scored if the drawing was done correctly.

Functional ability

This domain was assessed using information from a modified version of the Barthel Index which is one of the most widely-used assessments of functional independence[95]. Below we described the questions used in each country referring to functional ability.

Chile: 1. Has the subject had difficulty running or jogging a mile? 2. Has the subject had difficulty walking several streets? 3. Has the subject had difficulty sitting for two hours? 4. Has the subject had difficulty getting up from a chair? 5. Has the subject had difficulty climbing several floors? 6. Has the subject had difficulty climbing a floor? 7. Has the subject had difficulty bending over? 8. Has the subject had difficulty extending the arms? 9. Has the subject had difficulty pulling or pushing a large object? 10. Has the subject had difficulty lifting or carrying a weight of 5 kg? 11. Has the subject had difficulty lifting a coin off a table? 12. Does the subject have difficulty crossing the room? 13. Does the subject have difficulty dressing? 14. Does the subject have difficulty bathing? 15. Does the subject have difficulty eating? 16. Does the subject have difficulty lying down? 17. Does the subject have difficulty using a toilet? 18. Does the subject have difficulty preparing food? 19. Does the subject have difficulty handling money? 20. Does the subject have difficulty going places? 21. Does the subject have difficulty purchasing food? 22. Does the subject have difficulty making phone calls? 23. Does the subject have difficulty performing light work? 24. Does the subject have difficulty performing heavy work? 25. Does the subject have difficulty taking medicine? Uruguay: 1. Has the subject had difficulty running or jogging a mile? 2. Has the subject had difficulty walking several streets? 3. Has the subject had difficulty sitting for two hours? 4. Has the subject had difficulty getting up from a chair? 5. Has the subject had difficulty climbing several floors? 6. Has the subject had difficulty bending over? 7. Has the subject had difficulty extending the arms? 8. Has the subject had difficulty pulling or pushing a large object? 9. Has the subject had difficulty lifting or carrying a weight of 5 kg? 10. Has the subject had difficulty lifting a coin off a table? 11. Does the subject have difficulty crossing the room? 12. Does the subject have difficulty dressing? 13. Does the subject have difficulty bathing? 14. Does the subject have difficulty eating? 15. Does the subject have difficulty lying down? 16. Does the

subject have difficulty using a toilet? 17. Does the subject have difficulty preparing food? 18. Does the subject have difficulty handling money? 19. Does the subject have difficulty going places? 20. Does the subject have difficulty purchasing food? 21. Does the subject have difficulty making phone calls? 22. Does the subject have difficulty performing light work? 23. Does the subject have difficulty performing heavy work? 24. Does the subject have difficulty taking medicine? Ecuador: 1. Does the subject have difficulty running or jogging a mile or 15 blocks? 2. Does the subject have difficulty sitting for two hours? 3. Does the subject have difficulty getting up from a chair after sitting for a long time? 4. Does the subject have difficulty climbing several floors without resting? 5. Does the subject have difficulty bending, kneeling or stooping? 6. Does the subject have difficulty extending the arms above the shoulders? 7. Does the subject have difficulty pulling, pushing, or removing a large object, such as an armchair? 8. Does the subject have difficulty lifting or carrying a weight of more than 5 kilograms (10 pounds), such as a heavy shopping bag? 9. Does the subject have difficulty lifting a coin off a table? 10. Does the subject have difficulty crossing the room? 11. Does the subject have difficulty dressing (including putting shoes and socks)? 12. Does the subject have difficulty bathing (including getting in and out of the bathtub)? 13. Does the subject have difficulty eating (including cutting food, filling glasses)? 14. Does the subject have difficulty lying down or getting out of bed? 15. Does the subject have difficulty using a toilet (including sitting down and getting up)? 16. Does the subject have difficulty preparing hot food? 17. Does the subject have difficulty handling money? 18. Does the subject have difficulty going places alone? 19. Does the subject have difficulty purchasing food? 20. Does the subject have difficulty making phone calls? 21. Does the subject have difficulty performing light work? 22. Does the subject have difficulty performing heavy work? 23. Does the subject have difficulty taking medicine? Colombia: 1. Was the subject able to eat without assistance today? 2. Was the

subject able to bathe without assistance today? 3. Was the subject able to dress without assistance today? 4. Was the subject able to wash hands and face, comb his/her hair, shave or brush his/ her teeth without assistance today? 5. Is the subject a continent in terms of deposition? 6. Is the subject continent in terms of urination? 7. Was the subject able to use the toilet without assistance today? 8. Is the subject able to move from bed to chair? 9. Is the subject able to walk without assistance (independently)? 10. Is the subject able to go up and down stairs without assistance? , 11. Is the subject able of managing his/her money? 12. Is the subject able to shop on a daily basis? 13. Is the subject able of preparing food? Is the subject able to manage his/her medications? 14. Is the subject able to use public transportation? 15. Is the subject able of using the phone? 16. Does the subject have difficulty walking 5 blocks? 17. Does the subject need assistance to walk? 18. Is the subject able to walk 3 times a week between 9 and 20 blocks? 19. Is the subject able to walk 3 times per week less than 8 blocks?

Risk factors assessed in cross-sectional analyses

Demographics

Age

Chile/Uruguay/Ecuador/Colombia: The subject age in years.

Sex

Chile/Uruguay/Ecuador/Colombia: The subject's sex.

Social Determinants of health (SDH)

Education

Chile: 1. Elementary education, 2. Secondary education, 3. Primary education as a requirement, 4. Secondary education as a requirement, 5. Preparatory, 6. Normal, 7. Professional, 8. Postgraduate.

Uruguay: 1. Elementary education, 2. Secondary education, 3. Primary education as a requirement, 4. Secondary education as a requirement, 5. Preparatory, 6. Normal, 7. Professional, 8. Postgraduate. Ecuador: 1. None, 2. Literacy Center, 3. Kindergarten, 4. Elementary education, 5 Secondary education, 6. High School Cycle, 7. Postgraduate. Colombia: 1. None, 2. Incomplete elementary education, 3. Complete elementary education, 4. Incomplete Secondary education, 5. Complete Secondary education, 6. Technician or technologist without degree, 7. Technician or technologist with degree, 8. University without degree, 9. University with degree, 10. Postgraduate without degree, 11. Postgraduate with degree.

Isolation

Chile/Uruguay: How many people live in your household? Ecuador: Does the subject currently live alone or in company? Colombia: With whom does the subject live?

SES:

Chile/Uruguay: 1. Does the house have electric light? 2. Does the house have piped water? 3. Does the house have drainage? 4. Does the house have sewer system? 5. Does the house have a cement, brick or mosaic wood floor? 6. Does the house have a kitchen? 7. Does the house use gas or electricity for cooking? 8. Does the house have a bathroom inside the house? 9. Does the house have a toilet with a water connection? Ecuador: 1. Does the house have electric light? 2. Does the house have piped water inside the house? 3. Does the house have water from the public water supply? 4. Does the house have sewerage? 5. Does the house use the public sewer system for sewage disposal? 6. Does the house have a planking, tile, brick or mosaic wood floor? 7. Does the house have a kitchen? 8. Does the house use gas or electricity for cooking? 9. Does the house have a exclusive bathroom inside the house? 10. Does the house have a toilet with a water connection? Colombia: 1. Does the house have a kitchen? 2. Does the house have a exclusive bathroom? 3.

Does the house have a predominant brick, stone or polished wood interior walls? 4. Does the house have a predominant brick, stone or polished wood exterior walls? 5. Does the house have a predominant carpet, marble, planking, tile, brick or mosaic wood floor? 6. Does the house have a predominant cement roof? 7. Does the house have electric light? 8. Does the house have natural gas connected to the public supply? 9. Does the house have aqueduct? 10. Does the house have sewer?

Health status

Hypertension

Chile/ Uruguay: Does the subject have high blood pressure? Ecuador/Colombia: Has a doctor or nurse ever diagnosed whether the subject has hypertension?

Diabetes

Chile/Uruguay: Does the subject have diabetes? Ecuador/Colombia: Has a doctor or nurse ever diagnosed whether the subject has diabetes?

Heart Disease

Chile/Uruguay: Has the subject had a heart attack? Ecuador: Has a doctor or nurse ever diagnosed the subject with a heart attack, coronary heart disease, angina, congestive heart disease or other heart problems? Colombia: Has a doctor or nurse ever diagnosed the subject with a heart attack, pre- heart attack or other heart problems?

Falls

Chile/ Uruguay/Ecuador/Colombia: Has the subject suffered falls in the last 12 months?

Mental health symptoms

Chile/Uruguay: Does the subject have a nerve problem? Ecuador: Has a doctor or nurse ever diagnosed the subject with any nervous or mental health symptoms (such as: anxiety, depression, memory loss, behavioral memory loss, behavioral changes, among others)? Colombia: Has a doctor or nurse ever diagnosed the subject with any nervous, mental health symptoms?

Lifestyle

Alcohol consumption

Chile/Uruguay/Ecuador: The subjects: 1. never drinks 2. drinks less than one day per week 3. drinks 1 day a week 4. drinks 2 to 3 days a week 5. drinks 4 to 6 days a week 6. drinks everyday. Colombia: The subjects: 1. never drinks 2. drinks less than one day per week 3. drinks 2 to 3 days a week 4. drinks 4 to 6 days a week 5. drinks everyday

Physical activity

Chile/Uruguay: Does the subject perform rigorous activities? Ecuador: Did the subject perform rigorous exercise or physical activity, such as sports, jogging, dancing, or heavy labor, at least three times per week, in the last 12 months? Colombia: Does the subject participate at least three times per week, in any sporting activity or exercise such as swimming, jogging, tennis, cycling, aerobics, gymnastics classes or other activities, which cause sweating or make the subject breathless?

Smoking status

Chile/Uruguay/Ecuador: 1. The subject currently smoke. 2. The subject no longer smoke. 3. The subject never smoked. Colombia: 1. The subject smoked before and currently smoke 2. The subject smoked before and no longer smoke. 3. The subject did not smoke before and currently smoke. 4. The subject never smoked.

Description of healthy aging measures and risk factors assessed in the longitudinal analyses

Healthy aging measures

Cognition (MMSE)

Costa Rica/China: This metric was computed on 13 points by adding the correct answers or actions of a 5-question test. The test contained the following questions: Question 1. Subjects tell the date. This question scored 1 point for each of the following correct answers: the day of the week, the day of the month, the month and year. Question 2. The interviewer names 3 objects and the subjects must remember them. This question scored 1 point for each object recalled. Question 3. The interviewer gives the subject a piece of paper to perform 3 actions. Performing each action correctly scored 1 point, the actions were: taking a piece of paper with the right hand, folding the paper in half with both hands and placing the paper on her/his lap. Question 4. Subjects remember the name of the previous 3 objects again. This question was scored the same as question 2.

Healthy Aging (Barthel)

Costa Rica: 1. Was the subject able to talk? 2. Does the subject have difficulty walking 20 blocks? 3. Does the subject have difficulty pushing objects? 4. Does the subject have difficulty lifting arms? 5. Does the subject have difficulty cutting toenails? 6. Does the subject have difficulty preparing meals? 7. Does the subject have difficulty managing money? 8. Does the subject have difficulty shopping? 9. Does the subject have difficulty taking meds? China: 1. Does the subject require help for dressing? 2. Does the subject require help for washing face and hair and brushing teeth? 3. Does the subject require help for bathing and showering? 4. Does the subject require help for eating food that they have prepared? 5. Does the subject require help for getting in/out bed and walking across a room? 6. Does the subject require help for using the toilet? 7. Does the subject

require help using the toilet without spilling out urine? 8. Does the subject require help for grooming? 9. Does the subject require help for household chores? 10. Does the subject require help for preparing meals? 11. Does the subject require help for doing the laundry? 12. Does the subject require help going near places without transportation? 13. Does the subject require help going out using transportation? 14. Does the subject required help shopping? 15. Does the subject required help managing money? 16. Does the subject require help making and talking a call? 17. Does the subject required help taking

Risk factors assessed in longitudinal analyses

Demographics

Age

Costa Rica/China: The subject age in years.

Sex

Costa Rica/China: The subject's sex.

Social Determinants of Health

Education:

Costa Rica: 1. None, 2. Elementary education, 3. High School/technical, 4. Higher education/college. China: 1. Elementary school, 2. Middle school, 3. High School, 4. College/University.

Isolation

Costa Rica: Number of household members. China: This question was not in the survey.

Health Status

Diabetes

Costa Rica/China: Does the subject have diabetes?

Hypertension

Costa Rica: During the last 2 years, did a physician tell the subject that he/she has high blood pressure (hypertension)? China: Does the subject have hypertension?

Heart Disease

Costa Rica: During the last 2 years has a physician told the subject that he/she have had a heart attack? China: Since the previous interview, has the subject been diagnosed with a heart-related disease?

Falls

Costa Rica: This question was not in the survey. Kora: Has the subject been injured by a fall experience during the past 2 years?

Mental Health symptoms

Costa Rica: In the last 2 years has a physician told the subject that he/she has a nervous or mental health symptoms such as depression? China: Does the subject have emotional, nervous or mental health symptoms?

Lifestyle

Alcohol consumption:

Costa Rica: 1. The subject drinks occasionally, 2. The subject drinks daily, 3. The subject drinks in special occasions only, 4. Does the subject not currently drink?, 5. The subject has never drunk. China: 1. The subject never drinks, 2. The subject drinks normally, 3. The subject drinks excessively 4. The subject drinks severely (Alcoholic).

Physical activity

Costa Rica: In the last 12 months, did the subject exercise regularly or do other rigorous physical activities like sports, jogging, dancing, or heaving work, 3 times per week? China: Does the subject exercise regularly?

Smoking status:

Costa Rica: Does the subject currently smoke? China: 1. Did the subject smoke before and does the subject currently smoke? 2. Did the subject smoke before and does the subject no longer smoke? 3. Did the subject not smoke before and does the subject currently smoke? 4. Did the subject never smoke?
