# Supplementary material for: Factors associated with healthy aging in Latin American populations
Source: Nat Med. 2023 Aug 10;29(9):2248–58. doi: 10.1038/s41591-023-02495-1 (PMC10504086; doi:10.1038/s41591-023-02495-1)
Supplement: Supplementary file 2 — Reporting Summary [file 41591_2023_2495_MOESM2_ESM.pdf]

nature portfolio

Corresponding author(s): Agustin Ibanez and Hernando Santamaria-Garcia

Last updated by author(s): 2023/06/30

Reporting Summary

Nature Portfolio wishes to improve the reproducibility of the work that we publish. This form provides structure for consistency and transparency in reporting. For further information on Nature Portfolio policies, see our Editorial Policies and the Editorial Policy Checklist.

Statistics

For all statistical analyses, confirm that the following items are present in the figure legend, table legend, main text, or Methods section.

n/a	Confirmed	
☐	☒	The exact sample size (*n*) for each experimental group/condition, given as a discrete number and unit of measurement
☐	☒	A statement on whether measurements were taken from distinct samples or whether the same sample was measured repeatedly
☐	☒	The statistical test(s) used AND whether they are one- or two-sided<br>*Only common tests should be described solely by name; describe more complex techniques in the Methods section.*
☐	☒	A description of all covariates tested
☐	☒	A description of any assumptions or corrections, such as tests of normality and adjustment for multiple comparisons
☐	☒	A full description of the statistical parameters including central tendency (e.g. means) or other basic estimates (e.g. regression coefficient) AND variation (e.g. standard deviation) or associated estimates of uncertainty (e.g. confidence intervals)
☐	☒	For null hypothesis testing, the test statistic (e.g. *F*, *t*, *r*) with confidence intervals, effect sizes, degrees of freedom and *P* value noted<br>*Give P values as exact values whenever suitable.*
☒	☐	For Bayesian analysis, information on the choice of priors and Markov chain Monte Carlo settings
☐	☒	For hierarchical and complex designs, identification of the appropriate level for tests and full reporting of outcomes
☐	☒	Estimates of effect sizes (e.g. Cohen's *d*, Pearson's *r*), indicating how they were calculated

Our web collection on statistics for biologists contains articles on many of the points above.

Software and code

Policy information about availability of computer code: Data collection Data were collected using paper-pencil formats and REDCAP platforms following specific procedures in each country. The researchers were blinded to experimental condition and/or the study hypothesis during data collection.

Data analysis All analyses were run using Python version 3.9.13. All code for data analysis of this manuscript is available for download at GitHub at https//github.com/AI-BrainLat-team/Heterogeneous-risk-factors-LAC.

For manuscripts utilizing custom algorithms or software that are central to the research but not yet described in published literature, software must be made available to editors and reviewers. We strongly encourage code deposition in a community repository (e.g. GitHub). See the Nature Portfolio guidelines for submitting code & software for further information.

Data

Policy information about availability of data

All manuscripts must include a data availability statement. This statement should provide the following information, where applicable:

- Accession codes, unique identifiers, or web links for publicly available datasets
- A description of any restrictions on data availability
- For clinical datasets or third party data, please ensure that the statement adheres to our policy

Data availability: Data included in this study in the cross-sectional and longitudinal analyses were collected in the context of the National Aging surveys of five LACs countries (Ecuador, Colombia, Chile, Uruguay, and Costa Rica) and from China. Data from the National Aging Survey from Ecuador included in the study are publicly available and can be accessed after providing researchers information at this link: https// www.ecuadorencifras.gob.ec/encuesta-de-salud-bienestar-del-adulto-mayor/. Data from the National Aging Survey of Colombia (National Study of Health, Wellbeing, and Aging SABE Colombia 2015) can be accessed after fulfilling a registration form on the webpage of the Ministry of Health and Social Protection (Spanish: el Ministerio de Salud y de Protección Social) in Colombia. This procedure lasts around ten days and can be made at this link: https//www.datos.gov.co. Data from Chile and Uruguay included in this study was part of the SABE - Survey on Health, Wellbeing, and Aging in Latin America and the Caribbean, 2000 (ICPSR 3546). Data from this study is publicly available after including researchers' information in this link: https//www.icpsr.umich.edu/web/NACDA/studies/3546/versions/V1. Data from Costa Rica included in this study was taken from the Costa Rican Longevity and Healthy Aging Study (CRELES). Data from this study is publicly available after introducing researchers' information in this link: http//creles-download.demog.berkeley.edu/CRdata.pl.
Data from the National Aging Survey of China (China Health and Retirement Longitudinal Study (CHARLS)) can be accessed after researchers' registration at this link: https//charls.pku.edu.cn/en/.
Access to raw data from all databases described in this section can last around ten days.
The raw data generated in this study are also available for download at https//github.com/AI-BrainLat-team/Heterogeneous-risk-factors-LAC. All authors had access to the raw data. All software used in this study and its versions are specified on the condae_nv.yml file on the GitHub repository.

Human research participants

Policy information about studies involving human research participants and Sex and Gender in Research.

Reporting on sex and gender	In this study we have included sex and gender of participants in our plan of analyses.
Population characteristics	We ran two types of analyses. First, we implemented cross-sectional analyses in N=31,680 older adults (above of 60 years-old; female= 16,074; mean age=70.81, SD=8.22). Participants were recruited from the National Surveys on Health, Well-Being, and Aging performed in four LACs: Chile (2016, N=1,301; females=689; mean age=71.83 SD= 8,29), Uruguay (2011, N= 1,450; females=798; mean age=70.75 SD= 7,40), Ecuador (2012, N=5,235; females= 1,530; mean age= 70.09, SD= 7.82), and Colombia (2015, N=23,694; females=1,357; mean age=70.79 SD= 8.26).<br>In a second group of analyses, we implemented longitudinal analyses using data from the Longitudinal National aging study of Costa Rica, (Costa Rican Study on Longevity and Healthy Aging (CRELES), N=5,694, mean age=59.5, SD=3.2). Moreover, as control information, we also analyzed National Aging longitudinal information from China (assessing data of the China Health and Retirement Longitudinal Study (CHARLS), N=3,546, mean age=81,2, SD=10,1).
Recruitment	Participants were selected following a probabilistic, clustered, stratified, and multistage design in each country. We only included individuals with no diagnosis of dementia, as assessed during an initial screening. Databases of national surveys from all countries are open and were obtained following the established procedures for each country.
Ethics oversight	The Institutional Review Boards of Universidad Javeriana, Bogotá, Colombia (code FM773-2021) reviewed and approved the current study.

Note that full information on the approval of the study protocol must also be provided in the manuscript.

Field-specific reporting

Please select the one below that is the best fit for your research. If you are not sure, read the appropriate sections before making your selection.

☐ Life sciences ☒ Behavioural & social sciences ☐ Ecological, evolutionary & environmental sciences

For a reference copy of the document with all sections, see nature.com/documents/nr-reporting-summary-flat.pdf

Behavioural & social sciences study design

All studies must disclose on these points even when the disclosure is negative.

Study description	This study combine cross-sectional and longitudinal approaches and machine learning techniques on quantitative data taken from National Aging Surveys from four South American countries (Colombia, Ecuador, Chile, Uruguay), one Center American country (Costa Rica) and one Asian Country (China)
Research sample	The study included older individuals (above 60 years old, n=44,394 participants, females= 19,074; mean age=70.81, SD=8.22) randomly recruited from four South American countries (Colombia, Ecuador, Chile, Uruguay), one Center American country (Costa Rica), and one Asian Country (China). All datasets from the National Aging surveys from Latin American countries, in cross-sectional and longitudinal analyses, included representative samples from each country.
Sampling strategy	Individuals were included in this study following a probabilistic, clustered, stratified, and multistage approach implemented in the National Survey of Aging study in each country. No statistical method was used to predetermine sample size, considering we used the complete database of the National Aging surveys from different Latin American countries.
Data collection	Data were collected using paper-pencil formats and REDCAP platforms following specific procedures in each country. The researchers were blinded to experimental condition and/or the study hypothesis during data collection.
Timing	In general the start date of data collection was between 1999 and 2000 and the stop date was 2016. Moreover, data were collected differently in each country. In Colombia, data were recruited between 2014 to 2015, and in Ecuador, Chile, and Uruguay, data were collected between 1999 and 2000. Data from Costa Rica were collected in 2012, and 2016. Data from China was collected in 2014, and 2016.
Data exclusions	All variables used in the analyses of this study had complete values in at least 80% of cases. Thus, we ran our analyses in a sample of 37, 259 participants from 44,394 individuals.
Non-participation	We only assessed information of individuals who participated and finished all questions and requirements from the National Surveys of each country.
Randomization	Participants were not allocated in experimental groups.<br>As part of the machine learning techniques we implemented, the models used in this work follow standard random training-test procedures.

Reporting for specific materials, systems and methods

We require information from authors about some types of materials, experimental systems and methods used in many studies. Here, indicate whether each material, system or method listed is relevant to your study. If you are not sure if a list item applies to your research, read the appropriate section before selecting a response.

Materials & experimental systems

n/a	Involved in the study
☒	☐ Antibodies
☒	☐ Eukaryotic cell lines
☒	☐ Palaeontology and archaeology
☒	☐ Animals and other organisms
X	Clinical data
☒	☐ Dual use research of concern

Methods

n/a	Involved in the study
☒	☐ ChIP-seq
☒	☐ Flow cytometry
☒	☐ MRI-based neuroimaging

Clinical data

Policy information about clinical studies

All manuscripts should comply with the ICMJE guidelines for publication of clinical research and a completed CONSORT checklist must be included with all submissions.

Clinical trial registration	*Provide the trial registration number from ClinicalTrials.gov or an equivalent agency.*
Study protocol	*Note where the full trial protocol can be accessed OR if not available, explain why.*
Data collection	*Describe the settings and locales of data collection, noting the time periods of recruitment and data collection.*
Outcomes	*Describe how you pre-defined primary and secondary outcome measures and how you assessed these measures.*
